# A potential strategy against clinical carbapenem-resistant* Enterobacteriaceae*: antimicrobial activity study of sweetener-decorated gold nanoparticles in vitro and in vivo

**DOI:** 10.1186/s12951-023-02149-x

**Published:** 2023-11-06

**Authors:** Haifeng Liu, Zeyu Huang, Huanchang Chen, Ying Zhang, Pingting Yu, Panjie Hu, Xiaotuan Zhang, Jianming Cao, Tieli Zhou

**Affiliations:** 1 Department of Clinical Laboratory, The First Affiliated Hospital of Wenzhou Medical University, Wenzhou, Zhejiang China; 2 Key Laboratory of Clinical Laboratory Diagnosis and Translational Research of Zhejiang Province, Wenzhou, Zhejiang China; 3 School of Laboratory Medicine and Life Science, Wenzhou Medical University, Wenzhou, Zhejiang China

**Keywords:** Non-caloric artificial sweeteners, Gold nanoparticles, Carbapenem-resistant *Enterobacteriaceae*, Antimicrobial agents, Antibiofilm

## Abstract

**Background:**

Carbapenem-resistant *Enterobacteriaceae* (CRE) present substantial challenges to clinical intervention, necessitating the formulation of novel antimicrobial strategies to counteract them. Nanomaterials offer a distinctive avenue for eradicating bacteria by employing mechanisms divergent from traditional antibiotic resistance pathways and exhibiting reduced susceptibility to drug resistance development. Non-caloric artificial sweeteners, commonly utilized in the food sector, such as saccharin, sucralose, acesulfame, and aspartame, possess structures amenable to nanomaterial formation. In this investigation, we synthesized gold nanoparticles decorated with non-caloric artificial sweeteners and evaluated their antimicrobial efficacy against clinical CRE strains.

**Results:**

Among these, gold nanoparticles decorated with aspartame (ASP_Au NPs) exhibited the most potent antimicrobial effect, displaying minimum inhibitory concentrations ranging from 4 to 16 µg/mL. As a result, ASP_Au NPs were chosen for further experimentation. Elucidation of the antimicrobial mechanism unveiled that ASP_Au NPs substantially elevated bacterial reactive oxygen species (ROS) levels, which dissipated upon ROS scavenger treatment, indicating ROS accumulation within bacteria as the fundamental antimicrobial modality. Furthermore, findings from membrane permeability assessments suggested that ASP_Au NPs may represent a secondary antimicrobial modality via enhancing inner membrane permeability. In addition, experiments involving crystal violet and confocal live/dead staining demonstrated effective suppression of bacterial biofilm formation by ASP_Au NPs. Moreover, ASP_Au NPs demonstrated notable efficacy in the treatment of *Galleria mellonella* bacterial infection and acute abdominal infection in mice, concurrently mitigating the organism's inflammatory response. Crucially, evaluation of in vivo safety and biocompatibility established that ASP_Au NPs exhibited negligible toxicity at bactericidal concentrations.

**Conclusions:**

Our results demonstrated that ASP_Au NPs exhibit promise as innovative antimicrobial agents against clinical CRE.

**Graphical Abstract:**

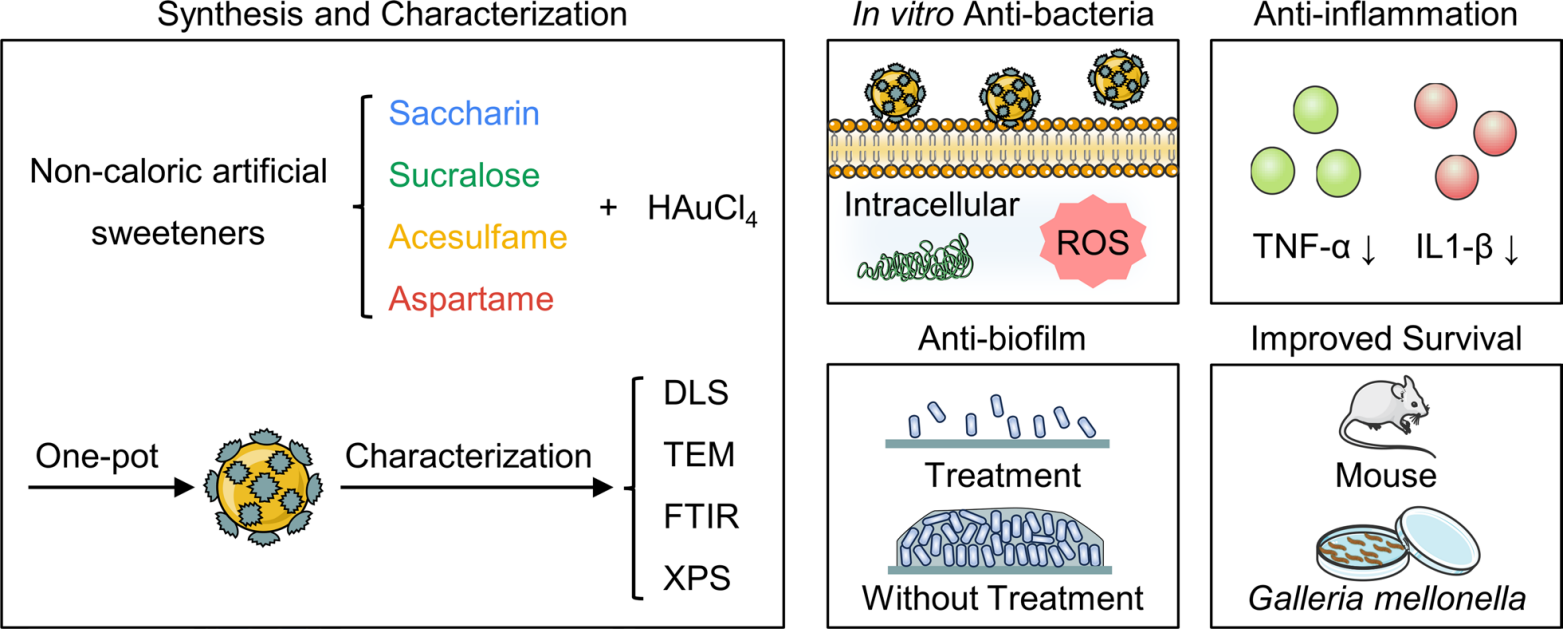

**Supplementary Information:**

The online version contains supplementary material available at 10.1186/s12951-023-02149-x.

## Introduction

The progression of bacterial antibiotic resistance is guiding humanity towards an era beyond antibiotics, where bacteria might eventually develop resistance to all antibiotics, rendering them ineffective. This scenario reminds us of the unsettling pre-antibiotic era [1]. An article published in The *Lancet* in 2019 serves as a reminder that extensively drug-resistant bacteria have triggered a global public health crisis, resulting directly in at least 1.27 million deaths and indirectly causing nearly 13.7 million deaths [2]. Among lots of antibiotic-resistant bacteria, carbapenem-resistant *Enterobacteriaceae* (CRE) presents a substantial challenge for clinical anti-infection treatments. They have spread extensively worldwide, leading to an elevated usage of last-line drugs in clinical treatment, such as tigecycline, ceftazidime-avibactam, and colistin [3]. However, the clinical use of last-line drugs is limited by their drawbacks, and in recent years, several bacteria have already outpaced these last lines of defense [4–6]. Within the challenging landscape of treating CRE, the mortality rate linked with its infections remains considerably high. Babiker et al. compiled clinical data on CRE infections at a local hospital from 2000 to 2017, revealing a 90-day mortality rate of up to 38% for CRE bloodstream infections [7]. This underscores the urgency of researching new antimicrobial agents or alternative treatment strategies to combat CRE.

At present, the prevailing strategies to address this issue involve the exploration of new drugs, encompassing the development of novel antibiotics and the decoration of existing substances. Additionally, combination therapy utilizing multiple antibiotics or non-antibiotics is being investigated to combat bacteria [8–11]. Combination therapy offers time-saving advantages but grapples with challenges related to solubility and cross-resistance, as many hydrophobic drugs necessitate dimethyl sulfoxide (DMSO) for dissolution [12], which could be detrimental to human health [13]; thus, limiting its advancement. The development of new antibiotics is a time-intensive process, and bacteria can swiftly evolve new resistance mechanisms, as seen with ceftazidime-avibactam, where strains with β-lactamase-related amino acid substitutions emerged within a few years of its introduction [14, 15], prompting a shift towards the development of less susceptible drugs.

Over time, nanotechnology has emerged and gained widespread application [16]. In the realm of nanomedicine, metal nanoparticles like silver, gold, zinc, and titanium decorated by some substances have exhibited remarkable antimicrobial and antibiofilm effects, with low in vivo toxicity [16–18]. Notably, there is scant literature reporting bacterial resistance to metal nanoparticles. These nanoparticles combat bacteria through mechanisms involving the generation of reactive oxygen species (ROS), membrane disruption, DNA degradation, and ATP depletio [19–21]. Nanoparticles with low resistance potential offer robust tools for addressing clinical resistance challenges [22].

Among these, gold nanoparticles (Au NPs) find wide-ranging applications due to their minimal toxicity, straightforward surface properties, and the potential for carrying the nanodecoration of existing substances [23]. Some literature has reported that Au NPs decorated with plant extracts or antibiotics exhibit excellent antimicrobial activity [24–26]. However, these substances might be constrained by extraction difficulties, low solubility, or limited applicability. Consequently, attention shifts to the environmentally friendly and widely applicable realm of the food industry, specifically non-caloric artificial sweeteners (NAS). NAS contain many hydroxyl and amino or amide groups, which have strong reducibility and are highly suitable for green synthesis of metal nanoparticles [27]. These common food additives, consumed globally by millions [28], are generally considered sugar substitutes that do not contribute to weight gain or elevated blood sugar levels [29]. Nevertheless, the safety of NAS at various doses remains a topic of intense debate, with evidence still lacking [30–33]. Among the numerous reports on NAS, four—saccharin (SAC), sucralose (SUC), acesulfame (ACE), and aspartame (ASP)—exhibit potential antimicrobial activity through ROS generation and alterations in membrane permeability [34]. However, their antimicrobial activity is exceedingly weak (minimum inhibitory concentration (MIC) > 1500 µg/mL) and unsuitable for biomedical applications. This forms the theoretical foundation and inspiration for our research. To date, very few studies have explored the decoration of Au NPs with NAS to investigate their antimicrobial properties. One article reported the antimicrobial activity of ASP-gold-silver hybrid nanoparticles (MIC ≥ 25 µg/mL) against *Escherichia coli* [35]. However, its synthesis process is excessively intricate, and the antimicrobial activity is attributed to the properties of the silver nanoparticles. In contrast, our study employs a simpler and more environmentally friendly method to produce ASP-decorated Au NPs (ASP_Au NPs) with lower toxicity and enhanced efficacy. Additionally, the biomedical applications of NAS are currently limited to weight reduction and obesity prevention [36], and their potential in the biomedical field requires further exploration. Given these circumstances, we believe that this research holds significant implications for both the food and medical industries.

## Results

### Synthesis and characterization of different Au NPs

The synthesis of Au NPs through a green one-pot method is a widely accepted and frequently employed approach known for its environmentally friendly, uncomplicated, and cost-effective characteristics [37]. This method principally involves combining tetrachloroauric acid (HAuCl_4_) with a reducing agent within the same container to produce organic or inorganic Au NPs [38]. The standard approach for generating Au NPs employs sodium borohydride (NaBH_4_) as the reducing agent [39]. Previous investigations have highlighted that reducible functional groups like hydroxyl, amino, and amide groups in organic compounds can effectuate the reduction of HAuCl_4_, yielding decorated Au NPs exhibiting varied functionalities [40]. In our study, we observed that NAS share similar chemical structures.

**Fig. 1:**
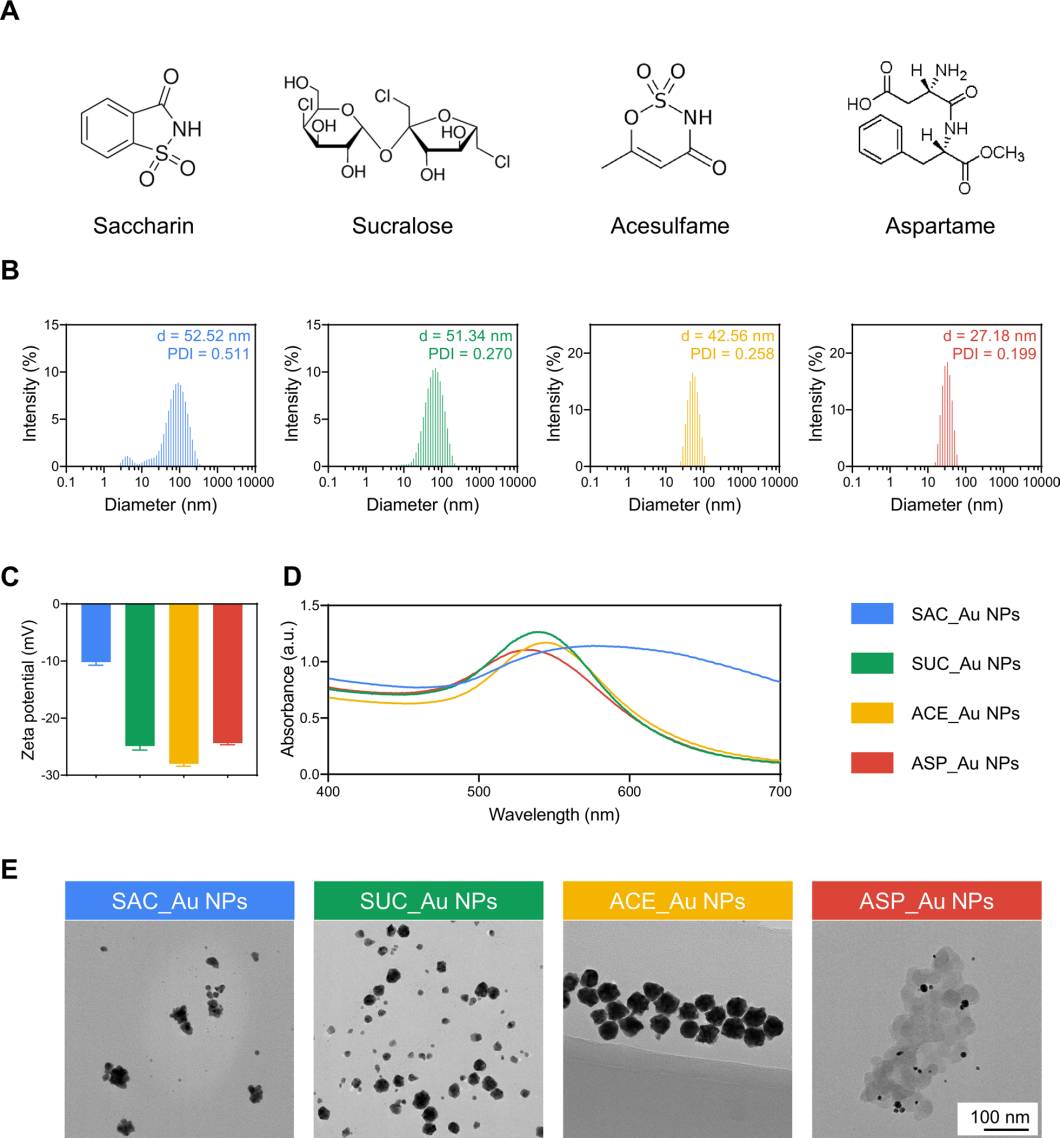
Characterization of different Au NPs. **A** Molecular structures of SAC, SUC, ACE, and ASP. **B** Particle size and dispersity of SAC_Au NPs, SUC_Au NPs, ACE_Au NPs, and ASP_Au NPs. **C** Zeta potential of SAC_Au NPs, SUC_Au NPs, ACE_Au NPs, and ASP_Au NPs. **D** UV–Vis spectra of SAC_Au NPs, SUC_Au NPs, ACE_Au NPs, and ASP_Au NPs. **E** TEM images of SAC_Au NPs, SUC_Au NPs, ACE_Au NPs, and ASP_Au NPs

In this study, we synthesized SAC-decorated Au NPs (SAC_Au NPs), SUC-decorated Au NPs (SUC_Au NPs), ACE-decorated Au NPs (ACE_Au NPs), and ASP_Au NPs by combining SAC (0.05 mmol), SUC (0.05 mmol), ACE (0.05 mmol), ASP (0.05 mmol) (Fig. 1A), and HAuCl_4_ (0.05 mmol). Due to the distinctive reducibility of NAS, the Au^3+^ ions in HAuCl_4_ undergo reduction to Au^0^ through Au-O or Au–N bonds, facilitating the formation of the aforementioned decorated Au NPs. Residual NAS within the system underwent removal through dialysis. Dynamic light scattering (DLS) measurements revealed that SAC_Au NPs, SUC_Au NPs, ACE_Au NPs, and ASP_Au NPs exhibited average sizes of 52.52, 51.34, 42.56, and 27.18 nm, respectively, with polydispersity indices (PDIs) of 0.511, 0.270, 0.258, and 0.199 (Fig. 1B). This suggests the presence of small and uniformly dispersed sizes among the four Au NPs. As depicted in Fig. 1C, SAC_Au NPs, SUC_Au NPs, ACE_Au NPs, and ASP_Au NPs displayed average zeta potentials of − 10.2, − 24.9, − 28, and − 24.4 mV, respectively, indicative of negatively charged nanoparticles with commendable stability. The UV–visible spectra exhibited absorption peaks within the 500–600 nm range, corresponding to the distinctive surface plasmon resonance of Au NPs (Fig. 1D). The UV–visible spectra of the Au NPs reduced by NaBH_4_ are shown inAdditional file 1: Figure S1.The noticeable redshift in the absorption peaks of the four NAS-decorated Au NPs (NAS_Au NPs) compared to those of the NaBH_4_-reduced Au NPs, which confirms the successful decoration of NAS on the gold nanoparticles [41]. The size, PDI, and zeta potential of NaBH4-reduced Au NPs are shown inAdditional file 1: Figure S2. Transmission electron microscopy (TEM) images (Fig. 1E) unveiled uniform morphology and reduced particle sizes for SAC_Au NPs, SUC_Au NPs, ACE_Au NPs, and ASP_Au NPs, approximately measuring 30, 28, 35, and 12 nm, respectively. The TEM sizes proved smaller than the DLS sizes, as DLS reflects hydrodynamic size while TEM evaluates the size of dry particles [42]. Notably, smaller-sized Au NPs have demonstrated enhanced antimicrobial activity [43], and ASP_Au NPs emerging as the standout among the four Au NPs due to their stability, well-defined circular shape, minimal size, and uniform dispersion. Further, the extent of NAS bound to the AuNPs was measured using the ortho-pthaldehyde (OPA) fluorescence assay [44]. As shown in Additional file 1: Table S1, it appears that approximately half of the NAS have bound to the Au NPs. OPA reacts with primary amines to produce fluorescence, and the lower binding efficiency observed may be due to some NAS participating in the reduction process, while others are involved in conjugation. This could result in alterations to the amino nitrogen groups, preventing their interaction with OPA. Additionally, we prepared standard Au NPs utilizing the NaBH_4_ reduction method for subsequent experimental control.

For in vitro antimicrobial activity, antibiofilm effects, biocompatibility, and in vivo model studies, the concentrations of SAC_Au NPs, SUC_Au NPs, ACE_Au NPs, and ASP_Au NPs were kept consistent with those of the Au NPs control group. Considering the challenges in determining post-dialysis concentrations of SAC, SUC, ACE, and ASP, their concentrations were maintained at levels similar to those of the pre-dialysis SAC_Au NPs, SUC_Au NPs, ACE_Au NPs, and ASP_Au NPs groups. In all experiments, the Au NPs control group refers to the group of NaBH_4_-reduced Au NPs.

### Antimicrobial activity of four NAS_Au NPs

As depicted in Additional file 1: Table S2, we selected 30 unique clinical CRE strains and 2 standard strains assessed their drug resistance profiles using the microbroth dilution technique. Polymerase chain reaction (PCR) and sequencing methodologies were employed to elucidate their resistance mechanisms, certain mechanism data were drawn from our prior studies [45, 46]. The chosen strains exhibited resistance mechanisms spanning all four classes of carbapenemases [47], encompassing Ambler class A (KPC, SHV, TEM, CTX-M, etc.), class B (NDM, IMP, etc.), class C (AmpC, CMY, etc.), and class D (OXA). Additional resistance mechanisms, such as efflux pumps, OmpK37 mutations, and downregulation of OmpC and OmpF, were also implicated. Overall, these strains displayed pronounced resistance to ertapenem (ETP) (≥ 64 µg/mL). Accordingly, we selected ETP as the antibiotic control for subsequent experiments, maintaining its concentration consistent with that of the ASP_Au NPs group to underscore the clinical applicability of our formulated Au NPs in addressing CRE-related challenges.

As delineated in Table 1, the MIC values of SAC_Au NPs, SUC_Au NPs, and ACE_Au NPs against the 32 tested bacterial strains all exceeded ≥ 256 µg/mL, signifying a lack of notable antimicrobial activity. Strikingly, ASP_Au NPs demonstrated exceptional antimicrobial potency against all strains, displaying MIC values ranging from 8 to 16 µg/mL, except for CG1330, which exhibited an MIC value of 4 µg/mL. The remarkable antimicrobial efficacy of ASP_Au NPs vis-à-vis the other three types of Au NPs may be attributed to their uniform particle shape, diminutive size, and the presence of ester, methyl, and phenyl moieties, which confer increased hydrophobicity. Enhanced hydrophobicity facilitates the internalization of particles into bacterial cells [48]. Considering ASP_Au NPs as a member of the NAS_Au NPs group with exceptional antimicrobial effects, we designated them for further characterization and subsequent exploration encompassing antimicrobial activity, antimicrobial mechanisms, antibiofilm activity, biocompatibility, anti-inflammatory properties, and in vivo antimicrobial assessments.

**Table 1:** Antimicrobial susceptibility of the SAC_Au NPs, SUC_Au NPs, ACE_Au NPs and ASP_Au NPs against the 32 strains used in this study

Species	Strains	MIC (μg/mL)			
		SAC_Au NPs	SUC_Au NPs	ACE_Au NPs	ASP_Au NPs
*E. coli*	DC2003	≥ 256	≥ 256	≥ 256	8
	DC5113	≥ 256	≥ 256	≥ 256	8
	DC5128	≥ 256	≥ 256	≥ 256	8
	DC5293	≥ 256	≥ 256	≥ 256	8
	DC6856	≥ 256	≥ 256	≥ 256	8
	DC7114	≥ 256	≥ 256	≥ 256	8
	DC7706	≥ 256	≥ 256	≥ 256	8
	DC8647	≥ 256	≥ 256	≥ 256	8
	DC10694	≥ 256	≥ 256	≥ 256	16
	DC11722	≥ 256	≥ 256	≥ 256	16
	ATCC25922	≥ 256	≥ 256	≥ 256	8
*K. pneumoniae*	FK2836	≥ 256	≥ 256	≥ 256	8
	FK3006	≥ 256	≥ 256	≥ 256	8
	FK3020	≥ 256	≥ 256	≥ 256	8
	FK6709	≥ 256	≥ 256	≥ 256	16
	FK6724	≥ 256	≥ 256	≥ 256	16
	FK7079	≥ 256	≥ 256	≥ 256	16
	FK7112	≥ 256	≥ 256	≥ 256	16
	FK7513	≥ 256	≥ 256	≥ 256	8
	FK8696	≥ 256	≥ 256	≥ 256	16
	FK9283	≥ 256	≥ 256	≥ 256	8
	ATCC700603	≥ 256	≥ 256	≥ 256	16
*E. cloacae*	CG648	≥ 256	≥ 256	≥ 256	16
	CG1038	≥ 256	≥ 256	≥ 256	8
	CG1181	≥ 256	≥ 256	≥ 256	8
	CG1212	≥ 256	≥ 256	≥ 256	16
	CG1249	≥ 256	≥ 256	≥ 256	8
	CG1257	≥ 256	≥ 256	≥ 256	8
	CG1330	≥ 256	≥ 256	≥ 256	4
	CG1381	≥ 256	≥ 256	≥ 256	8
	CG1737	≥ 256	≥ 256	≥ 256	8
	CG1813	≥ 256	≥ 256	≥ 256	16

### Further characterization of ASP_Au NPs

**Fig. 2:**
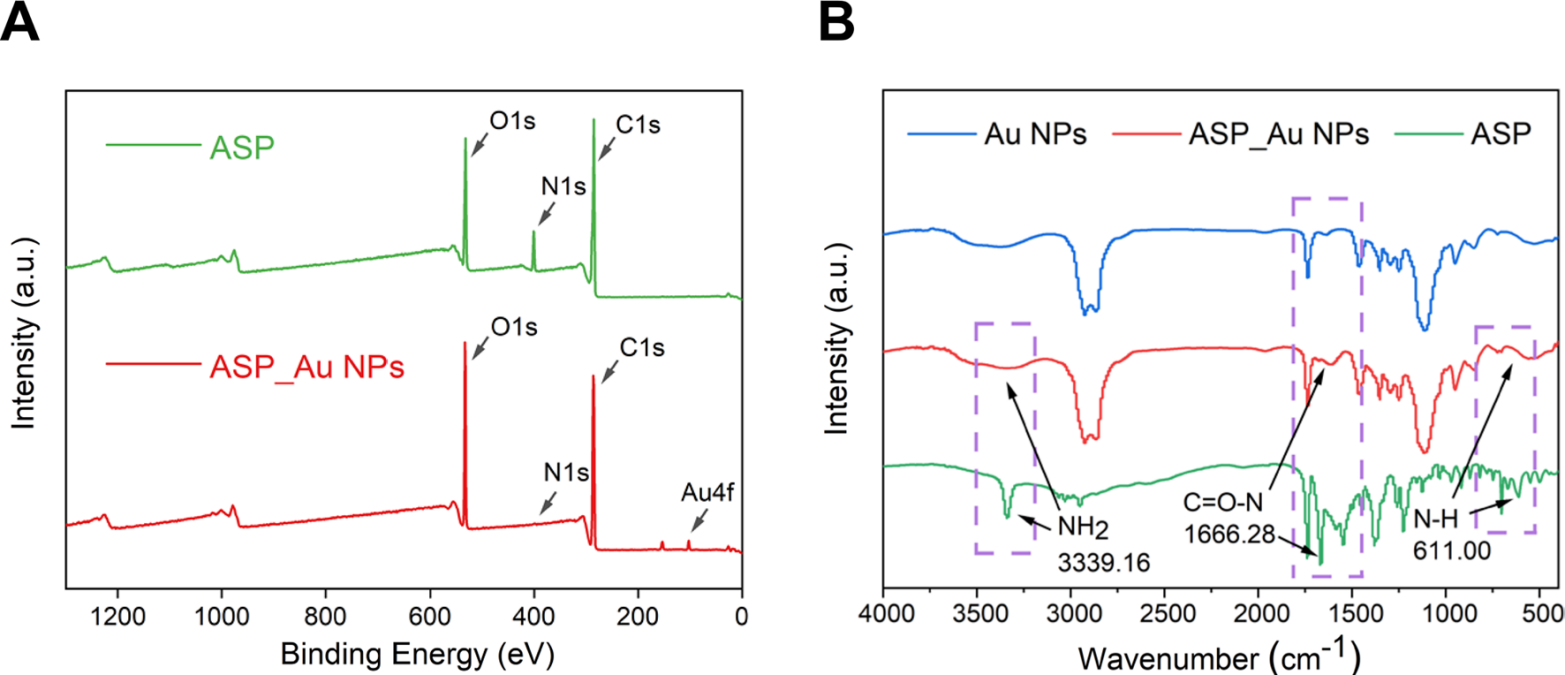
Further characterization of ASP_Au NPs.** A** XPS spectra of ASP and ASP_Au NPs.** B** FTIR spectra of ASP, Au NPs, and ASP_Au NPs

Given the potential of ASP_Au NPs in addressing clinical CRE challenges, we conducted supplementary characterization through X-ray photoelectron spectroscopy (XPS) and Fourier-transform infrared spectroscopy (FTIR) to gain deeper insights into ASP_Au NPs. As demonstrated in the XPS survey spectrum (Fig. 2A), ASP exhibited three discernible absorption peaks at approximately 530 eV, 400 eV, and 288 eV, correlating with the O1s, N1s, and C1s spectra, respectively. In contrast, ASP_Au NPs manifested three prominent absorption peaks at approximately 530 eV, 288 eV, and 87 eV, corresponding to the O, C, and Au elements, respectively. A comparison of the two spectra unveiled the disappearance of the N element peak and the amplification of the Au element peak in ASP_Au NPs, suggesting the plausible interaction between ASP's amino and amide groups with Au, leading to the establishment of Au–N bond connections and consequently causing the obliteration of the absorption peaks that were initially attributed to the amino and amide groups in ASP. To substantiate this hypothesis, we subsequently executed fitting of the Au 4f, N1s, and O1s spectra to scrutinize specific electron transfers during the chemical reaction (Additional file 1: Figure S3). The Au 4f spectra (Additional file 1: Figure S3A, B) exhibited two distinct absorption peaks for both ASP_Au NPs and standard Au NPs reduced by NaBH_4_, denoting Au 4f_5/2_ and Au 4f_7/2_. In view of the database and binding energy positions, these peaks indicated the presence of Au^0^ formation. Additionally, ASP_Au NPs displayed a feeble absorption peak at around 85.78 eV, suggesting marginal Au^1+^ formation, possibly attributable to electron transfer with -OH or nitrogen-containing groups. The N1s spectra (Additional file 1: Figure S3C, D) displayed two prominent absorption peaks for ASP located at 400.14 eV and 401.57 eV, attributed to –NH_2_ and –NH–, respectively. In ASP_Au NPs, two absorption peaks emerged at 397.06 eV and 399.78 eV, also corresponding to –NH_2_ and –NH–, implying a shift in the nitrogen-containing group’s peak pattern after loading Au onto ASP, signifying electron transfer between Au and N, leading to the establishment of an Au–N bond. The O1s spectra (Additional file 1: Figure S3E, F) unveiled conspicuous absorption peaks for ASP at 531.12 eV, 532.12 eV, and 533.88 eV, aligning with C = O, O = C–O, and C–OH, respectively. In comparison, ASP_Au NPs exhibited absorption peaks at 531.88 eV, 532.48 eV, and 532.92 eV, ascribed to C = O, O = C–O, and C–OH, respectively. The only notable shift was observed in the C–OH peak, suggesting electron transfer between C–OH and Au after Au loading, likely due to the emergence of a small quantity of Au^1+^.

The findings from FTIR analyses (Fig. 2B) indicated that the peak patterns of ASP_Au NPs resembled those of Au NPs. ASP displayed multiple absorption peaks, with a distinct peak at 1666.28 nm being the characteristic peak of ASP [49]. Following Au loading, ASP_Au NPs exhibited conspicuous shifts in absorption peaks at wavelengths of 611 nm, 1666.28 nm, and 3339.16 nm, attributed to N–H, C = O–N, and –NH_2_ [50]. The disappearance of these three peaks implied the reaction between ASP's amide structure (C = O–N–H) and amino group (–NH_2_) with Au, whereby the hydrogen on the nitrogen atom underwent substitution by Au to establish an Au–N bond, consistent with the XPS analysis. The distinct characteristic peaks of Au NPs, ASP_Au NPs, and ASP are presented in Additional file 1: Figure S4. FTIR of the remaining three nano-cargos are available in the Additional file 1: Figure S5.

### In vitro antimicrobial activity study of ASP_Au NPs

To investigate the antimicrobial activity of the raw materials, we conducted a microbroth dilution assay using the 32 bacterial strains. We evaluated the antimicrobial effects of ASP, Au NPs, and ASP_Au NPs separately. The results, as shown in Table 2, revealed that ASP and Au NPs alone had MIC values ≥ 256 µg/mL, indicating minimal antimicrobial activity as anticipated. However, ASP_Au NPs demonstrated remarkable antimicrobial potency. Although reports suggest that NAS shows some antimicrobial activity at concentrations of 1500 µg/mL and above [51], we believe that this concentration is much greater than the clinically used antibiotic doses and might cause certain side effects [52], making them unsuitable for use as antimicrobial agents.

**Table 2:** Antimicrobial susceptibility of the ASP, Au NPs, and ASP_Au NPs against the 32 strains used in this study

Species	Strains	MIC (μg/mL)		
		ASP	Au NPs	ASP_Au NPs
*E. coli*	DC2003	≥ 256	≥ 256	8
	DC5113	≥ 256	≥ 256	8
	DC5128	≥ 256	≥ 256	8
	DC5293	≥ 256	≥ 256	8
	DC6856	≥ 256	≥ 256	8
	DC7114	≥ 256	≥ 256	8
	DC7706	≥ 256	≥ 256	8
	DC8647	≥ 256	≥ 256	8
	DC10694	≥ 256	≥ 256	16
	DC11722	≥ 256	≥ 256	16
	ATCC25922	≥ 256	≥ 256	8
*K. pneumoniae*	FK2836	≥ 256	≥ 256	8
	FK3006	≥ 256	≥ 256	8
	FK3020	≥ 256	≥ 256	8
	FK6709	≥ 256	≥ 256	16
	FK6724	≥ 256	≥ 256	16
	FK7079	≥ 256	≥ 256	16
	FK7112	≥ 256	≥ 256	16
	FK7513	≥ 256	≥ 256	8
	FK8696	≥ 256	≥ 256	16
	FK9283	≥ 256	≥ 256	8
	ATCC700603	≥ 256	≥ 256	16
*E. cloacae*	CG648	≥ 256	≥ 256	16
	CG1038	≥ 256	≥ 256	8
	CG1181	≥ 256	≥ 256	8
	CG1212	≥ 256	≥ 256	16
	CG1249	≥ 256	≥ 256	8
	CG1257	≥ 256	≥ 256	8
	CG1330	≥ 256	≥ 256	4
	CG1381	≥ 256	≥ 256	8
	CG1737	≥ 256	≥ 256	8
	CG1813	≥ 256	≥ 256	16

**Fig. 3:**
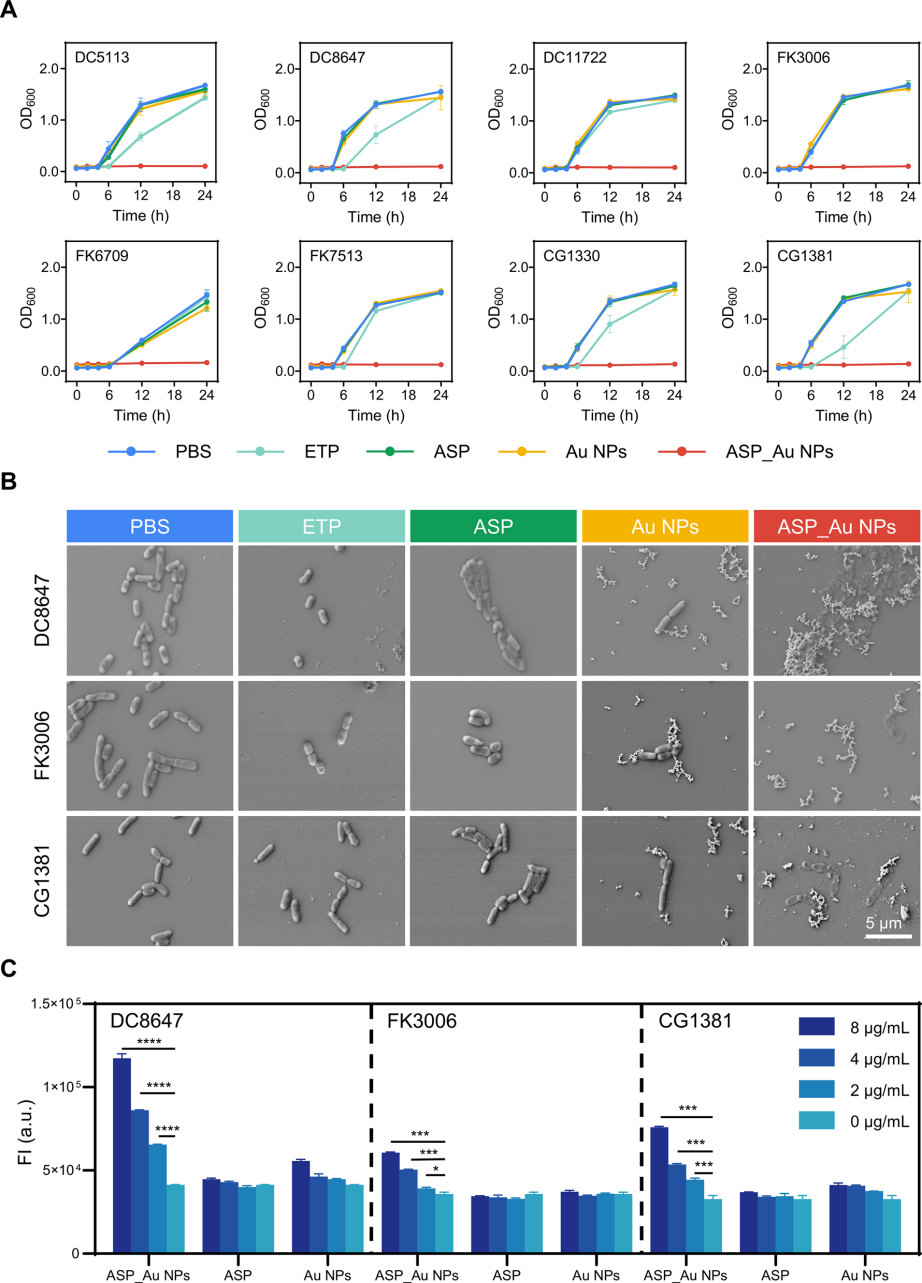
The antimicrobial ability of ASP_Au NPs in vitro and its core mechanism. **A** Growth curves of clinical CRE strains after treatment with different formulations. **B** Representative SEM images of clinical CRE strains after treatment with different formulations. **C** ROS levels in clinical CRE strains after treatment with different concentrations of formulations

For further insights into the effects of ASP_Au NPs on CRE strain growth kinetics, selected strains underwent growth curve analysis. As depicted in Fig. 3A, strains treated with phosphate-buffered saline (PBS), ETP, ASP, and Au NPs displayed normal growth, while ASP_Au NPs treatment led to effective antimicrobial action, preventing bacterial growth even after 24 h. The concentrations for each group were previously specified. Furthermore, to directly observe the morphological changes of bacteria after different treatments, a scanning electron microscopy (SEM) analysis was performed, and DC8647, FK3006, and CG1381 were randomly selected as the experimental strains. The SEM images (Fig. 3B) revealed that bacteria treated with ASP_Au NPs at the MIC concentration exhibited flattened and wrinkled morphology, along with distorted and ruptured cell bodies. In contrast, the bacteria in the other treatment groups maintained regular and intact cellular structures. Elevated ROS levels and membrane permeability changes are potential antimicrobial mechanisms of nanomaterials [53]. This aligns with our hypothesis, prompting us to conduct related experiments. The ROS detection assay demonstrated that ASP_Au NPs significantly raised ROS levels in strains DC8647, FK3006, and CG1381. The ROS increase displayed a dose-dependent pattern and even induced substantial ROS elevation in bacteria below the MIC concentration (Fig. 3C). To confirm that ROS is the core antimicrobial mechanism of ASP_Au NPs, we further performed ROS clearance experiments to investigate whether ASP_Au NPs still have significant antimicrobial activity after clearing ROS. In this study, quercetin, a plant-derived natural flavonoid known for its powerful antioxidant activity and ROS scavenging properties [54, 55], was used as the ROS scavenger. After quenching ROS, the MIC values of ASP_Au NPs for the same bacterial strains increased from 4–16 µg/mL to ≥ 256 µg/mL, an MIC escalation of at least 16–64 fold (Additional file 1: Table S3). This confirmed that elevated ROS levels are the primary antimicrobial mechanism of ASP_Au NPs. Additionally, we investigated the impact on inner membrane permeability. In the propidium iodide (PI) membrane permeability assay (Additional file 1: Figure S6), ASP_Au NPs at various concentrations significantly increased membrane permeability compared to the PBS control group (represented as 0 µg/mL), as evidenced by the corresponding rise in fluorescence intensity. Furthermore, ASP_Au NPs at the MIC concentration (8 µg/mL) exhibited a sharp increase in fluorescence intensity, albeit to a lesser extent at sub-inhibitory concentrations, but still with significant statistical differences. This suggests that ASP_Au NPs can interfere with bacterial morphology and normal physiological functions. Conversely, Au NPs and ASP at different concentrations did not exhibit a notable increase in fluorescence intensity. This finding aligns with the observed changes in bacterial morphology under scanning electron microscopy. Therefore, membrane permeability could potentially serve as a secondary antimicrobial mechanism of ASP_Au NPs.

Moreover, to exclude potential synergistic effects between ASP and Au NPs that might impact the study, a checkerboard assay was conducted for validation. As depicted inAdditional file 1: Table S4, within the randomly chosen experimental strains, both the combination treatment group and the monotherapy group exhibited MIC values of ≥ 256 µg/mL. This suggests the absence of a significant synergistic effect over a broad range. This implies that the antimicrobial potency of ASP_Au NPs originated from the "decoration" of ASP onto Au NPs, rather than a mere "synergistic" effect.

### In vitro biofilm inhibition study of ASP_Au NPs

Bacterial biofilms are communities of bacteria attached to surfaces, providing internal bacteria with significantly greater antibiotic resistance, up to thousands of times more [56]. In the context of hospital infection control, biofilms formed on medical devices and wound surfaces greatly impact patient health [57]. Furthermore, within the food industry, microbial biofilms often develop on food packaging, posing risks to food safety [58, 59]. Thankfully, numerous studies have reported the antimicrobial biofilm activity of various nanoparticles [60]. Hence, our focus was to investigate whether the ASP_Au NPs we synthesized possess biofilm inhibition properties, using a biofilm inhibition assay with crystal violet staining. Simultaneously, we employed confocal live/dead staining to directly observe the viability of bacteria within the biofilm.

**Fig. 4:**
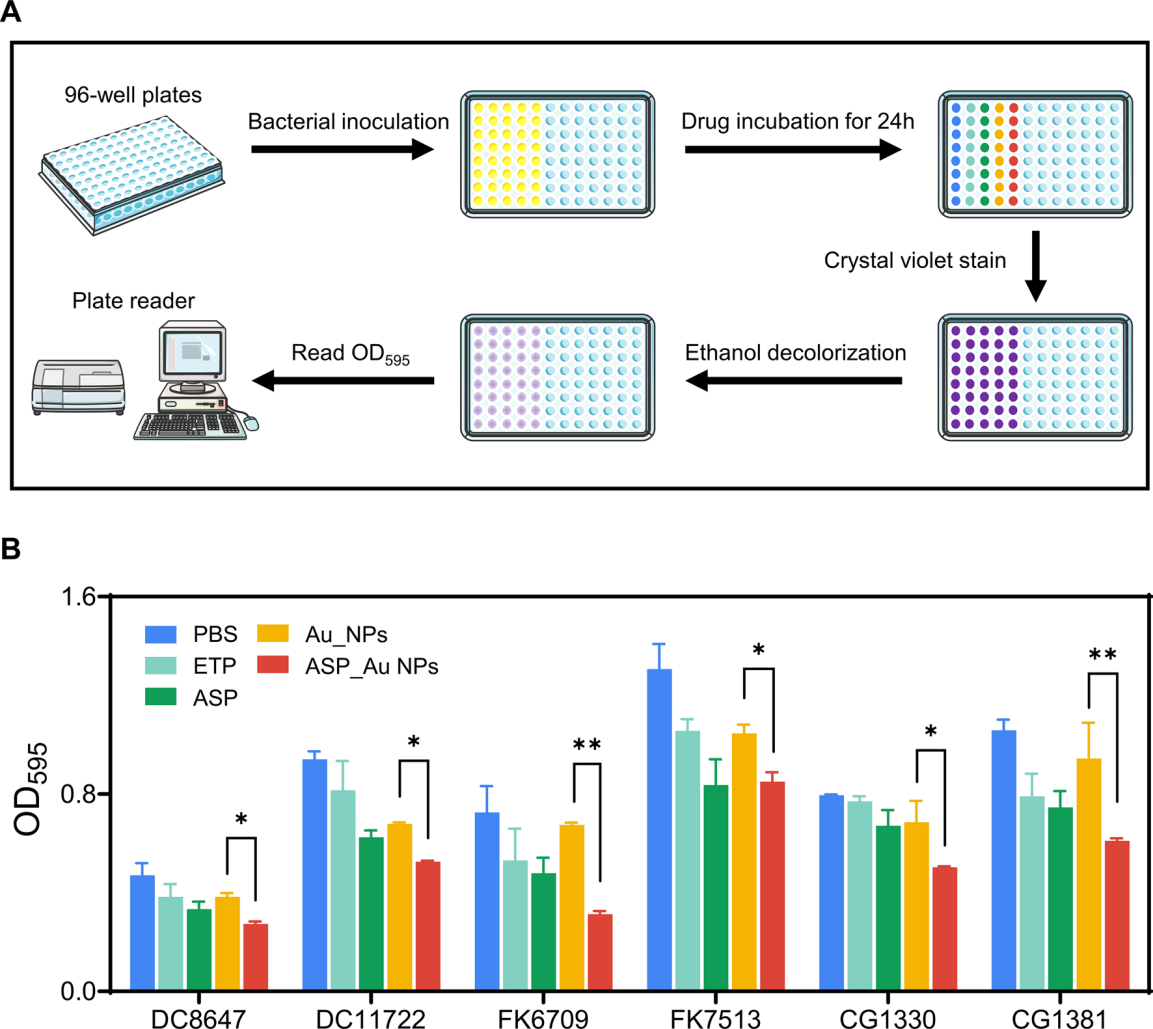
Inhibition of biofilm formation by ASP_Au NPs in vitro. **A** Schematic illustration of the crystal violet staining method to assess biofilm formation inhibition. **B** Crystal violet staining results at OD_595_ after treatment with different formulations in clinical CRE strains

In the biofilm inhibition assay, we selected six strains randomly as experimental subjects, and the concentration of ASP_Au NPs was determined based on 1/2 of the MIC. The concentrations of ETP, ASP, and Au NPs were specified earlier. Figure 4A outlines the general process of the biofilm inhibition assay. As depicted in Fig. 4B, the group treated with ASP_Au NPs significantly impeded biofilm formation, whereas the ETP and Au NPs groups did not exhibit this effect. Intriguingly, we also observed a slight inhibitory effect on biofilm formation by ASP. Regarding this observation, we found supporting evidence in the relevant literature that ASP has potential ability to inhibit biofilm formation [61, 62]. This implies that the potential ability of ASP_Au NPs to inhibit biofilm formation might stem from the decoration of ASP. Furthermore, confocal live/dead staining substantiated this observation. As depicted in Additional file 1: Figure S7, green fluorescence represents live bacteria, while red fluorescence indicates dead bacteria. At sub-inhibitory concentrations, ASP causes a slight reduction in bacterial density within the biofilm, while ASP_Au NPs are able to kill nearly half of the bacteria. Consequently, We contend that ASP_Au NPs exhibit superior antibiofilm activity compared to ASP and Au NPs because they can better penetrate bacterial biofilms and kill more bacteria, resulting in the inhibition of biofilm growth within the same timeframe.

### Safety assessment of ASP_Au NPs

**Fig. 5:**
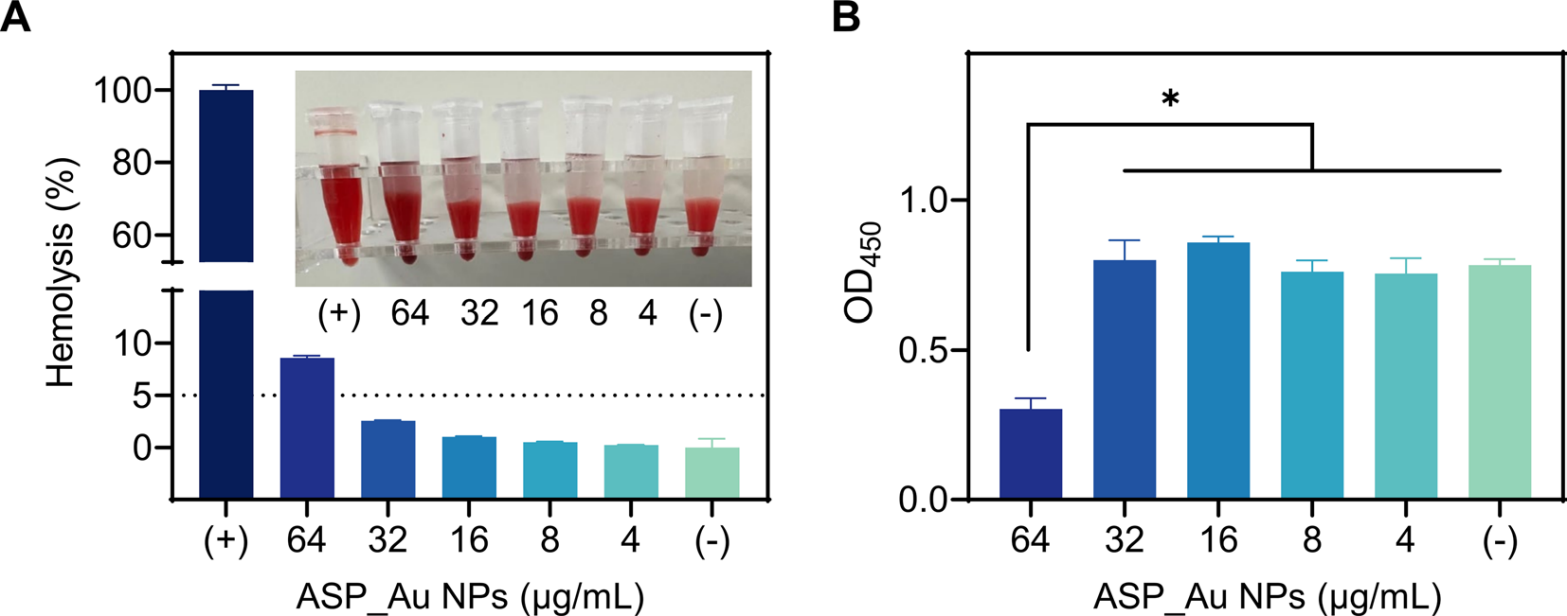
Safety evaluation of ASP_Au NPs. **A** Hemolytic effects of ASP_Au NPs at various concentrations. **B** Cell toxicity of ASP_Au NPs at different concentrations using the CCK-8 assay. Statistical differences in OD_450_ readings indicate cytotoxicity

In order to simulate in vivo safety and further explore the therapeutic effects, we conducted hemolysis and cytotoxicity assays. The hemolysis assay, depicted in Fig. 5A, demonstrated that ASP_Au NPs at concentrations 2–8 times the MIC (≤ 32 µg/mL) did not manifest any hemolytic activity, remaining below the defined cutoff of hemolysis rates ≤ 5%. For the cytotoxicity assay, the CCK-8 assay method was employed. As illustrated in Fig. 5B, exposure to ASP_Au NPs at concentrations 2–8 times the MIC (≤ 32 µg/mL) had no impact on cell viability. Collectively, ASP_Au NPs exhibited antimicrobial and antibiofilm effects without eliciting corresponding toxicity.

### In vivo antimicrobial performance study

**Fig. 6:**
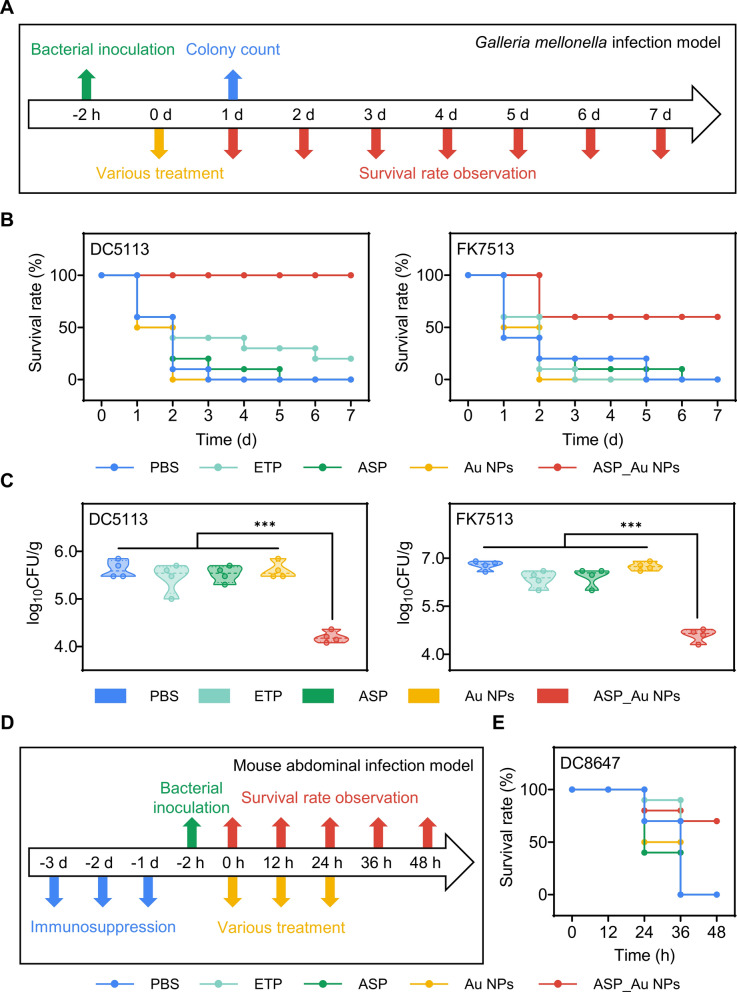
In vivo antimicrobial performance of ASP_Au NPs. **A** General experimental procedure of the *Galleria mellonella* bacterial infection model. **B** Survival of *Galleria mellonella* larvae infected with clinical CRE and treated with different formulations at different time points. **C** Bacterial load in *Galleria mellonella* larvae infected with clinical CRE and treated with different formulations after 24 h. **D** General experimental procedure of the acute intraperitoneal infection model in mice. **E** Survival of mice infected with clinical CRE and treated with different formulations at different time points

Building upon the remarkable in vitro antimicrobial efficacy and robust safety profile of ASP_Au NPs, we proceeded to investigate their effects within a *Galleria mellonella* infection model and a mouse acute intra-abdominal infection model. The *Galleria mellonella* infection model was chosen to validate the in vivo antimicrobial performance of ASP_Au NPs. Multiple authoritative studies have highlighted *Galleria mellonella*'s role as a stable in vivo model, characterized by its cost-effectiveness, user-friendliness, and absence of ethical limitations. This model produces results similar to those achieved through vertebrate in vivo experiments [63–65]. In our initial step, we introduced various concentrations of ASP_Au NPs into healthy *Galleria mellonella* to gauge in vivo safety and dismiss the possibility of drug-induced mortality. Illustrated in Additional file 1: Figure S8, all *Galleria mellonella* (10 per group) injected with ASP_Au NPs survived across the wide concentration range tested. Consequently, DC5113 and FK7513 were selected as the experimental strains to establish a CRE infection model. Adjustments were made to the methods according to prior studies [66]. The general experimental procedure is outlined in Fig. 6A. Succinctly, healthy *Galleria mellonella* weighing between 200–300 mg were distributed into five groups: PBS, ETP, ASP, Au NPs, and ASP_Au NPs, with 10 larvae per group. Bacterial suspensions (DC5113, 10 µL, 1.5 × 10^8^ CFU/mL; FK7513, 10 µL, 7.5 × 10^6^ CFU/mL) were microsyringe-injected into the penultimate left limb under consistent conditions. After a 2 h bacterial exposure, ASP_Au NPs were injected into the penultimate right limb at a concentration of 16 µg/mL, as determined from the MIC in the in vitro antimicrobial assay. The concentrations of ETP, ASP, and Au NPs remained as previously indicated. Larval survival was documented daily, with mortality ascertained in the absence of response to physical stimuli. As demonstrated in Fig. 6B, *Galleria mellonella* infected with DC5113 and treated with ASP_Au NPs exhibited a 100% survival rate, while those infected with FK7513 and treated with ASP_Au NPs showed a 70% survival rate. Conversely, larvae in the PBS, ETP, ASP, and Au NPs groups predominantly perished within 7 days. These findings underscored the notable enhancement in the survival rate of CRE-infected *Galleria mellonella* through ASP_Au NPs, aligning with mouse model results. Furthermore, bacterial colony counting was conducted to reflect the in vivo antimicrobial effects of ASP_Au NPs, with slight decorations based on bacterial strain characteristics [67]. Briefly, bacteria resistant to CRE strains were selectively isolated from *Galleria mellonella* a day after standard treatment. Following the euthanasia of four larvae per group, their bodies were processed, yielding a suspension in PBS. A 10 µL aliquot of the suspension was placed onto an Luria Bertani (LB) agar plate containing ETP, and visible colonies were tallied the following day. As depicted in Fig. 6C, the bacterial load in *Galleria mellonella* treated with ASP_Au NPs was markedly lower than that in the PBS, ETP, ASP, and Au NPs groups, for both DC5113 and FK7513 infections. The ASP_Au NPs treatment group witnessed nearly a 2 log_10_ CFU/g reduction in bacterial load, whereas no significant differences were noted in the other groups. These results affirm the remarkable in vivo antimicrobial properties of ASP_Au NPs.

Regarding the mouse acute intra-abdominal infection model, Fig. 6D outlines the comprehensive procedure for in vivo mouse experiments. We employed immunosuppressant cyclophosphamide (150 mg/kg) to induce immune-deficient mice, simulating individuals with compromised immune function. DC8647 was selected as the experimental strain. Following three consecutive intraperitoneal immunosuppressant injections, mice were inoculated with a bacterial suspension (200 µL, 1.5 × 10^8^ CFU/mL) supplemented with 5% yeast extract into the peritoneal cavity [68]. Given the frequent clinical application of carbapenems like ETP, an ETP control group was incorporated. The mice were divided into five groups—PBS, ETP, ASP, Au NPs, and ASP_Au NPs—each consisting of 10 mice. Previous reports informed the methods employed to construct mouse models in vivo [69]. After a 2 h bacterial exposure, the corresponding drugs were administered at 0 h, 12 h, and 24 h. ASP_Au NPs were administered at a concentration of 10 mg/kg, as previously described, through a 200 µL injection. Mouse survival was monitored every 12 h, alongside measurement of the mice's body weight. As revealed in Fig. 6E, mice treated with ASP_Au NPs showed a 70% survival rate, while those in the PBS, ETP, ASP, and Au NPs groups succumbed within 48 h. These results highlight the potential of ASP_Au NPs in countering CRE infections in vivo and significantly improving the survival rates of infected mice. Moreover, changes in mouse body weight were tracked as an indirect indicator of the drug's effects. As depicted in Additional file 1: Figure S9, mice treated with ASP_Au NPs exhibited a significantly slower decrease in body weight compared to those in the PBS, ETP, ASP, and Au NPs groups, underscoring the in vivo anti-infective effects of ASP_Au NPs. To conclude, both the mouse model and *Galleria mellonella* model corroborate the potential of ASP_Au NPs as a promising option against clinical CRE infections.

### In vivo biocompatibility assessment

In light of the demonstrated efficacy of ASP_Au NPs treatment in mice, an in-depth exploration of the inflammatory response and physiological alterations in mice was conducted to assess the physiological ramifications of ASP_Au NPs in vivo. The mouse model methodology was replicated, except for the administration of immunosuppressants and bacterial inoculation, with the objective of observing the impact of ASP_Au NPs on mouse immune response and physiological functions. Initially, histopathological sections were prepared from the heart, liver, spleen, lung, and kidney of mice that were sacrificed 48 h post-treatment. Hematoxylin and eosin (H&E) staining was carried out to evaluate the infiltration of inflammation in various organ tissues. As illustrated in Additional file 1: Figure S10, the organ tissue morphology in mice treated with ASP_Au NPs appeared normal and similar to the PBS group, with no marked indications of inflammation infiltration or tissue congestion. This suggests that ASP_Au NPs are nonhazardous to mouse organ tissues and do not elicit excessive inflammatory reactions.

Subsequently, blood samples were obtained via retro-orbital bleeding for comprehensive blood count analysis in order to monitor the differential leukocyte count. The outcomes, as shown in Additional file 1: Figure S11, indicated that the counts of white blood cells (WBC) and neutrophils (NEUT) in mice treated with ASP_Au NPs were comparable to those in the PBS group, with all values falling within the normal reference range (WBC: 0.8–10.6 10^9^/L; NEUT: 0.23–3.6 10^9^/L). This suggests that ASP_Au NPs did not lead to a significant rise in white blood cell count that could trigger an undue immune response. Additionally, an analysis of biochemical markers was performed by subjecting the blood samples to centrifugation and serum testing. This analysis focused on eight liver function markers, four cardiac enzyme markers, and three kidney function markers, all in line with clinical practice. As depicted in Additional file 1: Figure S12, all the biochemical markers in mice treated with ASP_Au NPs showed no notable disparities in comparison to the PBS group, with the exception of alkaline phosphatase (ALP) and creatinine (CREA) levels, which were below the reference range. Meanwhile, the other biochemical markers remained within the normal reference range, which is outlined in Additional file 1: Table S5. It is important to note that elevated ALP and CREA levels might imply impaired liver or kidney function. However, it usually holds no clinical significance when these markers fall below the reference range and might be attributed to methodological factors. In conclusion, ASP_Au NPs have demonstrated outstanding biocompatibility in vivo, positioning them as promising candidates for potential in vivo applications.

### Evaluation of anti-inflammatory effects

**Fig. 7:**
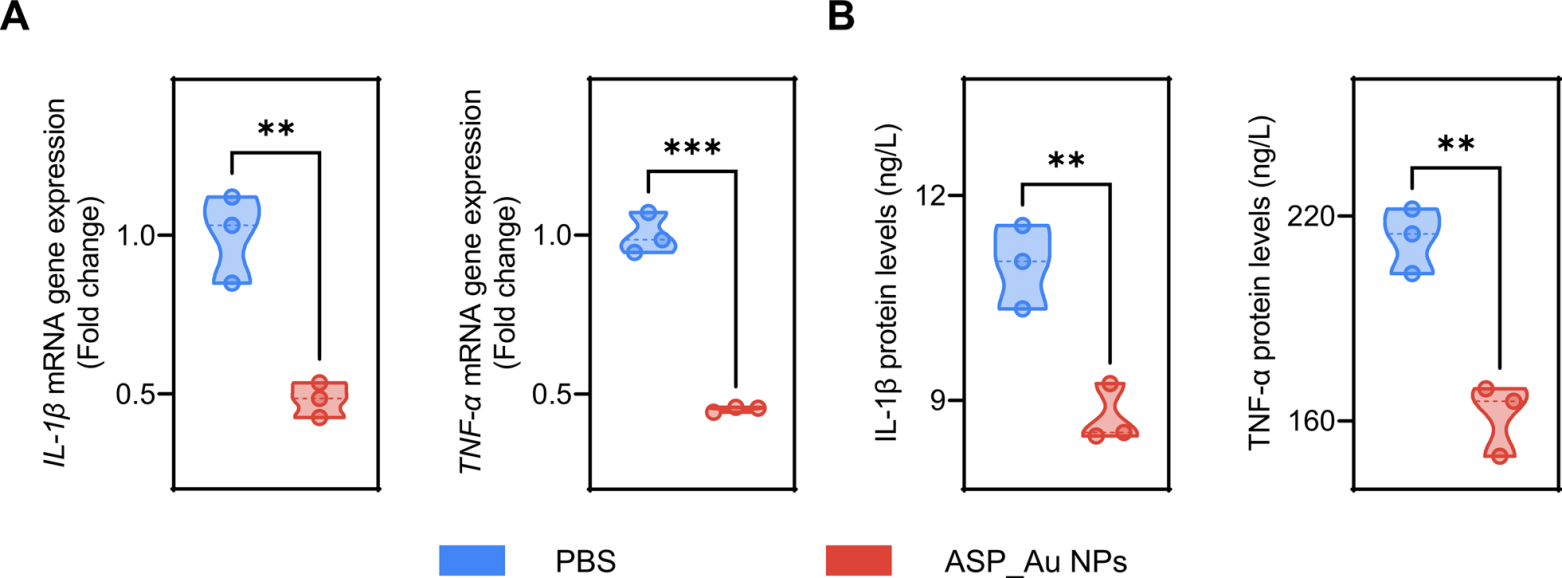
Evaluation of the anti-inflammatory performance of ASP_Au NPs. **A** Gene expression levels of *IL-1β* and *TNF-α* mRNA in cells treated with PBS and ASP_Au NPs measured by RT-qPCR. **B** Protein levels of IL-1β and TNF-α in cells treated with PBS and ASP_Au NPs detected using an ELISA assay

Commercially available NAS has shown specific anti-inflammatory effects [70]. As a NAS member, ASP also exhibits analgesic properties similar to nonsteroidal anti-inflammatory drugs, and it can diminish the levels of the inflammatory cytokine IL-6 [71]. Hence, the hypothesis was formulated that ASP_Au NPs could possess potential anti-inflammatory effects. To validate this hypothesis, real time quantitative PCR (RT-qPCR) and enzyme-linked immunosorbent assay (ELISA) were conducted. For the RT-qPCR experiment, an inflammatory response was triggered in RAW264.7 mouse macrophage cells in vitro utilizing lipopolysaccharide (LPS) [72]. The LPS-stimulated cells were divided into two groups: the PBS group and the ASP_Au NPs group. The ASP_Au NPs concentration was determined based on the MIC from the antimicrobial assay, which was 16 µg/mL. Following 4 h of drug treatment, cellular RNA was extracted for RT-qPCR analysis to quantify the relative expression levels of inflammatory cytokines. As depicted in Fig. 7A, the levels of *IL-1β* and *TNF-α* in the ASP_Au NPs group were significantly downregulated compared to the PBS group, indicating the potential anti-inflammatory effect of ASP_Au NPs. For the ELISA experiment, a similar approach to the RT-qPCR experiment was adopted, wherein IL-1β and TNF-α protein detection kits were utilized to measure the cytokine levels in the cell supernatant obtained after centrifugation. As shown in Fig. 7B, the protein levels of IL-1β and TNF-α in the ASP_Au NPs group were significantly diminished in comparison to the PBS group, aligning with the outcomes of the RT-qPCR analysis. Thus, our findings suggest that ASP_Au NPs may hold potential anti-inflammatory effects.

## Discussion

In light of the escalating bacterial resistance and widespread usage of carbapenem drugs [73], the defense has been breached by numerous bacteria, leading to grave infections attributed to CRE in clinical environments [74]. Many antibiotics are continuously being adapted by bacteria [75, 76]. Using β-lactam drugs as an example, bacteria have developed numerous enzyme-based resistance mechanisms. Sublethal antibiotic concentrations can facilitate the transfer of resistance genes [77, 78], posing a challenge for treating multidrug-resistant bacteria. A significant advantage of nanoparticles is their lower potential for resistance development. They employ distinct mechanisms from traditional antibiotics, either individually or in combination, to eliminate bacteria [79]. These mechanisms are also less prone to resistance development [80].

Moreover, nanoparticles can function as delivery systems, enabling antibiotics to penetrate barriers and exert their effects [81, 82]. They can also act as aggregating and stimulating agents, inducing local amplification and cumulative effects that cause bacteria to succumb to oxidative stress [83]. Currently, there is minimal evidence of bacteria developing resistance to nanoparticles. As a result, the low likelihood of resistance development to nanoparticles stands as a key driver behind their increasing popularity. Motivated by interdisciplinary strategies and the ascent of nanotechnology, we embarked on exploring and devising a novel prospective nanoantibiotic to tackle the urgent clinical antibiotic resistance dilemma. Current nanoantibiotics predominantly consist of metal-based nanoparticles, with silver nanoparticles being extensively studied [84–86], and widely employed [87]. However, silver nanoparticles have significant drawbacks, particularly their notable toxicity [88]. In contrast, Au NPs exhibit lower toxicity and photothermal effects, suggesting their more promising applications [89–91]. Therefore, we shifted our focus to Au NPs.

Existing approaches to decorate Au NPs as antimicrobial agents primarily involve plant extracts such as polyphenols, flavonoids, and traditional Chinese herbs [92, 93]. Alternatively, Au NPs can be loaded with antibiotics to reinstate the sensitivity of drug-resistant bacteria [94–96]. Additionally, chemical entities like amino acids, peptides, and extracellular polysaccharides have been coupled with Au NPs to augment their antimicrobial properties [97–99]. These studies provide a foundational understanding for applying nanotechnology in medicine. Nonetheless, it is apparent that these substances may harbor high toxicity, elevated costs, intricate synthesis processes, or limited applicability due to their underlying raw materials. These factors impose constraints on the application and evolution of these Au NPs. Hence, we chose not to pursue the trajectories of plant-based, antibiotic-based, or peptide-based methodologies. Instead, we opted to employ established and ecologically benign substances, such as NAS, for decorating Au NPs. Through a relatively straightforward and cost-efficient synthesis procedure, our aim was to achieve a certain degree of antimicrobial activity, thereby expanding their utilization as food additives or as antimicrobial agents to address drug-resistant bacteria in clinical settings, thus, contributing to the medical realm. The notion of constructing NAS_Au NPs was born from the fact that NAS itself harbors latent weak antimicrobial effects. However, realizing this effect requires significantly higher concentrations compared to the clinical administration of antibiotics, and high NAS concentrations may exert certain adverse effects on the human body, which has sparked controversy across various studies [100–102]. Consequently, the clinical application of NAS as an antimicrobial agent lacks substantial significance. Nevertheless, NAS finds broad utility, is cost-effective, and enjoys ready availability, ensuring its pragmatic worth. Thus, we harnessed extant nanotechnology to decorate Au NPs with NAS, with the aim of probing their potential in addressing the pressing issue of CRE.

To our astonishment, we unearthed that a constituent of NAS_Au NPs, namely ASP_Au NPs, exhibited excellent antimicrobial prowess at lower concentrations. Furthermore, the concentration requisite for antimicrobial activity stood lower than that of certain antibiotics [103], pointing to its clinical applicability. The hemolysis assay, cytotoxicity evaluation, and in vivo compatibility assays collectively underscored the safety of ASP_Au NPs, thereby signaling their suitability for in vivo employment. Though our assessment of inflammatory factors and concentrations in vitro was partial, lending insights into potential anti-inflammatory effects, our optimism persists. This can be further supported by the extant literature that corroborates the viability of ASP decoration for designing potential anti-inflammatory agents [104]. Our study lays a foundation for subsequent research and the formulation of fresh therapeutic avenues, such as employing ASP_Au NPs as adjuncts in treating drug-resistant bacterial wound infections. Of noteworthy significance, ASP_Au NPs demonstrated remarkable in vivo antimicrobial efficacy across both CRE-infected mouse and *Galleria mellonella* models, underscoring their potential in tackling drug-resistant bacterial infections. The fundamental antimicrobial mechanism of ASP_Au NPs hinges on dose-dependent amplification and accumulation of ROS, a phenomenon validated through ROS detection and clearance experiments, consonant with mechanisms underpinning some existing Au NPs-based antimicrobial agents [105]. The impact on bacterial inner membrane permeability represents a secondary antimicrobial mechanism of ASP_Au NPs, as confirmed in the PI membrane permeability assay. Negatively charged ASP_Au NPs can bind more effectively to the bacterial membrane, thereby enhancing membrane permeability, leading to the leakage of cellular contents and bacterial death. Furthermore, in contrast to the almost negligible antimicrobial efficacy of ASP alone (MIC ≥ 1500 µg/mL), ASP_Au NPs evinced antimicrobial activity at concentrations as low as 4–16 µg/mL. Through nanodecoration, the antimicrobial activity of ASP was increased by a factor of at least 94–375, rendering ASP_Au NPs with small particle size and stable morphology, thus, validating the success of our decoration of ASP.

Our investigation into clinical strains has unveiled a remarkable property of ASP_Au NPs: the capacity to inhibit biofilm formation. While ASP alone demonstrates limited antibiofilm capability, it does not exhibit the robust antimicrobial activity displayed by ASP_Au NPs. This could be attributed to the sub-inhibitory concentrations of ASP_Au NPs, which kill almost half of the bacteria within the biofilm, thus affecting biofilm formation, as observed in the confocal live/dead staining of the biofilm. This underscores the imperative of nanodecoration. Nanoparticles possessing dual antimicrobial and antibiofilm traits hold potential for applications like coatings for food packaging [106–109], indicating the diverse possibilities for employing ASP_Au NPs within the food industry. Notably, our research establishes a bridge between nanotechnology, medicine, and the food industry. The ASP_Au NPs we have synthesized may emerge as a versatile platform, especially considering the ongoing safety debates surrounding NAS [110–112]. By surmounting limitations and reducing ASP concentrations, ASP_Au NPs can extend their utility and forge a new avenue in nanotechnology research for clinical antimicrobial agents. Meanwhile, the evaluation of resistance development against ASP_Au NPs was not carried out, leaving it as a topic that will necessitate future investigation.

In summary, our research validates the potential of ASP_Au NPs as clinical antimicrobial agents. Furthermore, given the wide-ranging applications of ASP itself, ASP_Au NPs hold significant promise in the field of biomedical research. We believe that the utilization of nanotechnology to enhance existing substances holds immense potential and warrants further investigation. Nevertheless, our experiments have primarily revealed the novel antimicrobial traits of ASP and NAS. We have elucidated their fundamental antimicrobial mechanisms and assessed their in vivo safety and effectiveness. The nanosynthesis system proposed in this study may not be the most optimal, however, this represents one of the potential avenues for future in-depth research. Meanwhile, subsequent research and comprehensive in vivo or clinical trials are imperative to confirm whether ASP_Au NPs can indeed offer a solution to tackle the urgent issue of CRE.

## Conclusion

In the post-antibiotic era, the problem of CRE needs to be solved urgently. Decorating widely used substances through nanotechnology to transform commonly used substances into potential antimicrobial agents represents a captivating and profoundly significant avenue. Our study has extended the application scope of NAS, particularly with the utilization of ASP on a broader platform. ASP stands as the most prevalent food additive in current global use. Through nanodecoration, we have crafted ASP_Au NPs. The antimicrobial and anti-inflammatory prowess, coupled with the remarkable biocompatibility of ASP_Au NPs, positions them as promising contenders for forthcoming clinical applications. Additionally, their capability to inhibit biofilm formation hints at potential uses in the food industry, such as coatings for food packaging. The adoption of a straightforward, eco-friendly, one-step synthesis process further underscores the feasibility of large-scale ASP_Au NPs production. As components of the NAS_Au NPs family, ASP_Au NPs offer compelling and encouraging prospects. We hold the view that nanotechnology will persist as a driving force in interdisciplinary applications. In the future, more researchers are likely to use nanotechnology to decorate commonly employed substances or delve deeper into exploring the clinical utility of nanostructured antimicrobial agents.

## Materials and methods

All experiments involved in this study were repeated at least thrice.

### Materials

The key materials employed in this study, along with their respective manufacturers, were given in Additional file 1: Table S6.

### Bacterial strains

In this study, we employed 30 clinical CRE strains, encompassing 10 strains of *Escherichia coli*, 10 strains of *Klebsiella pneumoniae*, and 10 strains of *Enterobacter cloacae*. These strains were isolated from the First Affiliated Hospital of Wenzhou Medical University (Zhejiang, China). In addition, two standard strains—*Escherichia coli* ATCC 25922 and *Klebsiella pneumoniae* ATCC 700603—were procured from the NCCL. Matrix-assisted laser desorption/ionization time-of-flight mass spectrometry (MALDI-TOF/MS; bioMérieux, Lyon, France) was employed to identify all clinical strains. The isolated strains were cryopreserved in LB broth supplemented with 30% glycerol at –80 °C for subsequent investigations. Notably, the strains retained stable resistance even after repeated subculturing.

### Preparation and characterization of various Au NPs

The procedure for producing all Au NPs followed the outlined method with slight decorations [68]. In summary, NAS (including SAC, SUC, ACE, ASP; 0.05 mmol), Tween 80 (50 mg), and triethylamine (50 µL) were dissolved in 10 mL of ice-cold water using sonication (Instrument: DSA50-JY2, Frequency: 40,000 Hz, Power: 50 W) for 15 min, resulting in a well-dissolved mixture. Under vigorous stirring (1000 rpm) at room temperature, HAuCl_4_·3H_2_O (0.05 mmol, 400 µL) was gradually added to the mixture. Continuous stirring for an additional 2 h yielded Au NPs once the solution turned purple or wine red. For the removal of unreacted compounds, the Au NPs underwent dialysis against double-distilled water for 24 h, followed by sterilization through passage via a 0.22 µm filter. The dialysis filter membrane employed was MD55 (7000 D), with double-distilled water (ddH_2_O) used as the dialysis buffer. The dialysis membrane with this pore size effectively retained the Au NPs, while the residual substances were filtered into the buffer. A nanoparticle size and Zeta-potential analyzer (DLS; Malvern Zetasizer Nano ZS90, England) was employed to assess nanoparticle charge and dispersion. The UV–vis absorption of distinct Au NPs was measured using a multifunctional microplate reader (BioTek Synergy NEO_2_, America). Transmission electron microscopy (TEM; JEOL JEMF200, Japan) was utilized to characterize the morphology of different Au NPs. For ASP_Au NPs, additional structural characterization was conducted through XPS (Thermo Scientific K-Alpha, USA) and FTIR (Thermo Scientific Nicolet iS5, USA). For the OPA fluorescence assay, 8 mg of OPA reagent in 100 μL of 95% v/v ethanol was mixed with 200 µL of β-mercapto ethanol and 10 mL of 0.4 M boric acid titrated with NaOH to PH 9.7, as previously reported [44]. Next, equal volumes of OPA solution and either NAS or fully dialyzed NAS_Au NPs solutions were mixed, and the fluorescence emission at 455 nm (with excitation at 340 nm) was measured using a microplate reader. Eventually, the extent of NAS conjugated to the AuNPs was estimated by measuring the fluorescence of the supernatant before and after conjugation.

### Antimicrobial activity testing

The MICs of imipenem (IPM), meropenem (MEM), and ETP were determined in accordance with the guidelines of the Clinical and Laboratory Standards Institute (32nd edition). As previously delineated, the MIC values of various Au NPs, ASP, and standard Au NPs were measured individually [113]. Briefly, LB broth containing a bacterial density of 1.5 × 10^6^ CFU/mL was prepared beforehand. The formulations were serially diluted in sterile LB broth to achieve diverse concentrations, yielding a final volume of 100 µL. These diluted formulations were then combined with LB broth containing bacteria, culminating in a final volume of 200 µL. Subsequent incubation at 37 °C for 16–18 h facilitated visual observation of bacterial growth. The MIC was defined as the minimum concentration at which no discernible bacterial growth occurred [114].

### Growth curve

The growth curve experiment mirrored the antimicrobial susceptibility test. Initially, 200 µL of LB broth containing a bacterial density of 1.5 × 10^6^ CFU/mL was inoculated with varied concentrations of ETP, ASP, Au NPs, or ASP_Au NPs. A microplate reader was utilized to measure absorbance at 600 nm at 0, 2, 4, 6, 12, and 24 h.

### SEM for observation of bacterial morphology

To observe bacterial morphology, silicon chips (3 × 3 mm) were placed in a 24-well plate, providing a substrate for bacterial attachment. Subsequently, 1 mL of LB broth containing a bacterial density of 1.5 × 10^6^ CFU/mL and corresponding concentrations of ETP, ASP, Au NPs, or ASP_Au NPs were incubated at 37 °C for 24 h. The silicon chips were then rinsed thrice with PBS, fixed in 2.5% glutaraldehyde for 15 min at low temperature, and subjected to dehydration for 10 min using incrementally increasing ethanol concentrations (30%, 50%, 70%, 80%, 90%, and 100%). After air-drying, gold sputter-coating was performed, followed by SEM observation (Hitachi SU8010, Japan).

### ROS detection and ROS clearance

For ROS detection, a commercial kit was employed to quantify ROS production in bacteria, as previously outlined [115]. Single strains of *Escherichia coli*, *Klebsiella pneumoniae*, and *Enterobacter cloacae* were selected as experimental strains. Overnight LB bacterial cultures were washed thrice with PBS and diluted in PBS to achieve an OD_600_ of 0.3–0.4. Cells were incubated with the fluorescent probe 2′,7′dichlorodihydrofluorescein diacetate (DCFH-DA) at a 1:1000 ratio in the dark at 37 °C for 30 min. Following treatment with ASP, Au NPs, and ASP_Au NPs for 2 h, intracellular ROS production was quantified using 10 µM DCFH-DA. Regarding ROS clearance, as previously described [116], quercetin was employed as a ROS scavenger. Similar to the antimicrobial susceptibility test, quercetin was introduced to LB broth containing a bacterial density of 1.5 × 10^6^ CFU/mL, pre-treated with ASP_Au NPs, at a final concentration of 16 µg/mL. The mixture was incubated at 37 °C for 16–18 h, and bacterial growth was visually ascertained.

### Detection of inner membrane permeability by PI

The previously outlined method [12] underwent slight modification. Log-phase cultures of *Escherichia coli*, *Klebsiella pneumoniae*, and *Enterobacter cloacae* were exposed to varying concentrations of ASP, Au NPs, ASP_Au NPs, or PBS,for 2 h, and followed by treatment with 50 μg/mL PI for 30 min. Subsequently, the fluorescence intensity were recorded on a microplate reader at 561 nm.

### Checkerboard antimicrobial susceptibility test

The previously outlined method [117] underwent slight modification. In brief, diverse dilutions of ASP and Au NPs were separately and collectively administered to a bacterial density of 1.5 × 10^6^ CFU/mL. The mixture was then placed in incubation at 37 °C for 16–18 h, with visual observation of bacterial growth.

### Biofilm inhibition assay

The crystal violet staining approach, previously described with slight decorations [118], was employed. Bacteria at a density of 1.5 × 10^6^ CFU/mL were exposed to PBS, ETP, ASP, Au NPs, or ASP_Au NPs at the respective concentrations in 200 µL of LB medium. Following a 24-h incubation at 37 °C, the biofilms were gently washed and allowed to air-dry. Subsequently, 1.0% crystal violet (200 µL) was introduced to the biofilms. After a 15-min staining interval, redundant crystal violet was rinsed away, and the biofilms were permitted to air-dry. Next, 95% ethanol and 5% acetic acid (200 µL) were added to dissolve the crystal violet within the biofilms. The dissolved crystal violet was then transferred to a pristine 96-well plate, and the microplate reader was utilized to measure absorbance at 600 nm.

For confocal live/dead staining, the method described in the previous study [119] was used with minor modifications. We selected FK6709 as the experimental bacterial strain and examined the alterations in its biofilm following treatment with various drugs. In summary, FK6709 (10^6^ CFU/mL) was inoculated into LB broth containing sub-inhibitory concentrations of ASP_Au NPs, as well as the same concentration of ASP, Au NPs, ETP, or PBS. This mixture was placed in a six-well plate and incubated at 37 °C to allow for the formation of static biofilms on glass coverslips for 24 h. The biofilms were rinsed twice with sterile PBS to eliminate planktonic cells and subsequently subjected to live/dead staining using SYTO 9 and PI, following the prescribed procedure. Subsequently, the biofilms were washed twice with sterile PBS to remove any excess staining, and imaging of the biofilms was performed using laser confocal microscope (Nikon A1, Japan). The Z-axis height was determined by scanning from the point where fluorescence first appeared, representing the uppermost layer, to the point where fluorescence disappeared, signifying the lowermost layer. This process involved scanning across 20 layers in total.

### Hemolysis assay

The method and cutoff criteria outlined previously [120] underwent minor adjustments. Fresh mouse blood underwent three rounds of washing with physiological saline, following which red blood cells (RBCs) were extracted through centrifugation at 3000 rpm for 5 min. These RBCs were subsequently diluted with physiological saline to create a 5% RBC suspension. Simultaneously, ASP_Au NPs at varying concentrations were subjected to incubation with the RBC suspension at 37 °C. After centrifugation, the supernatant was transferred to a 96-well plate, and the microplate reader was utilized to measure absorbance at 545 nm. Hemolysis rate (%) was computed as (OD experimental group – OD negative control group)/(OD positive control group—OD positive control group). The negative control group was exposed solely to physiological saline, while the positive control group was subjected to 0.1% Triton X-100.

### Cell toxicity assay

The previously described method [121] was slightly modified. Cells utilized in the experiment were nurtured in Dulbecco's Modified Eagle Medium (DMEM) supplemented with 10% heat-inactivated fetal bovine serum (FBS) at 37 °C with 5% CO_2_ and 95% air within a humidified atmosphere. A total of 100 µL of RAW 264.7 cell suspension containing 10^5^ cells was introduced into each well of a 96-well plate. Subsequently, 10 µL of varying ASP_Au NPs concentrations or PBS was added to the medium. After a 16 h incubation, 10 µL of CCK-8 reagent was added to each well, followed by a 2 h incubation at 37 °C. The microplate reader was employed to measure absorbance at 450 nm.

### In vivo models

The study encompassed a mouse acute peritoneal infection model and a *Galleria mellonella* infection model. Pertaining to the *Galleria mellonella* model, the specific workflow is elaborated upon in the ‘‘Results’’ section. The criteria for selecting *Galleria mellonella* were formerly detailed [122]. In the context of the mouse acute peritoneal infection, 50 male ICR mice (6 to 8 weeks old, specific pathogen-free [SPF]) were procured from Vital River (Zhejiang, China). Ethical review and approval by the Ethics Committee of the First Affiliated Hospital of Wenzhou Medical University (Approval No.: SYXK 2021–0017) guided the animal research. All animal studies were executed in accordance with the Wenzhou Experimental Animal Welfare and Ethics Standards. The ‘‘Results’’ section offers insight into the specific workflow. Euthanasia was conducted on surviving mice upon the conclusion of the experiments.

### Biocompatibility

Healthy mice were divided into two groups, with 4 mice in each group, and randomly assigned to the PBS group and the ASP_Au NPs group. Injection dosage and frequency aligned with the treatment model of mouse acute peritoneal infection. At the 48 h mark, major organs (heart, liver, spleen, lung, and kidney) were collected from the mice and preserved in a 4% formaldehyde solution. The tissues were then embedded in paraffin, yielding 5 µm sections. H&E dye was employed for staining, and microscopy facilitated observation. Blood was also collected through retro-orbital bleeding at the 48-h juncture for hematological and biochemical parameter analyses, following the manufacturer's instructions. Automated hematology and biochemical analyzers (Mindray BC-2800vet, Chemray 240, Chemray 420, and Chemray 800) facilitated blood analysis.

### Inflammatory factor detection

Two experiments, namely RT-qPCR and ELISA, were conducted to detect inflammation factors. Concerning RT-qPCR, the method was executed as previously described [123] with slight decorations. In summary, LPS (1 µg/mL) was employed to simulate bacterial-induced cellular inflammation, followed by co-incubation of cells with PBS, ASP_Au NPs, and cells for a duration of 4 h. Total RNA was extracted from each group of 10^6^ cells using Trizol reagent, and then transferred to 1.5 mL EP tubes in accordance with the manufacturer's instructions (Biomiga, Shanghai, China). Subsequently, purified RNA was reverse transcribed into cDNA using the RevertAid First Strand cDNA Synthesis Kit, and amplification was conducted using TB Green Premix Ex Taq II (Tli RNaseH Plus). In the qPCR reaction, *β-actin* was utilized as the reference gene, and the relative expression levels of *IL-1β* and *TNF-α* were calculated employing the 2^–ΔΔCt^ method. The primer sequences employed in the RT-qPCR procedure are outlined in Additional file 1: Table S7. As for ELISA detection, the method was carried out as previously outlined [124] with slight decorations. In line with the manufacturer’s instructions, the IL-1β and TNF-α protein detection kits were employed. In essence, LPS (1 µg/mL) was used to emulate bacterial-induced cellular inflammation, followed by co-incubation of cells with PBS, ASP_Au NPs, and cells for a duration of 4 h. Following centrifugation (12,000 rpm, 5 min), the supernatant was collected, and subsequent steps were executed according to the kit instructions.

### Statistical analysis

Data were exhibited as mean ± standard deviation (SD) from at least three independent trials. Statistical analysis was performed using the Student's t-test or One-way analysis of variance (ANOVA), and *P* values < 0.05 were considered statistically significant (indicated with *), < 0.01 (indicated with **), < 0.001 (indicated with ***), and < 0.0001 (indicated with ****). GraphPad Prism 9.0 (GraphPad Software, LLC; San Diego, California, USA) software was used for statistical analysis.

### Supplementary Information

**Additional file 1: Figure S1.** UV-visible spectra of NaBH4-reduced Au NPs. Inside the dashed box, a comparison of the peaks between NaBH4-reduced Au NPs and NAS_Au NPs are presented. **Figure S2.** The size, PDI and zeta potential of NaBH4-reduced Au NPs. **Figure S3.** XPS analysis of Au 4f, N1s, and O1s for ASP, Au NPs, and ASP_Au NPs. **A** Au 4f spectrum analysis of Au NPs. **B** Au 4f spectrum analysis of ASP_Au NPs. **C** N1s spectrum analysis of ASP. **D** N1s spectrum analysis of ASP_Au NPs. **E** O1s spectrum analysis of ASP. **F** O1s spectrum analysis of ASP_Au NPs. Hollow dots represent raw data, blue curves represent the overall fitting curve of the data, and black curves represent the baseline. Colored curves and their corresponding peak labels are shown in the figure. **Figure S4.** FTIR peaks of Au NPs, ASP, and ASP_Au NPs. **Figure S5.** The FTIR spectra for the remaining three nano-cargos. **Figure S6.** PI membrane permeability assay. There was a sharp increase in fluorescence intensity at MIC concentrations (8 μg/mL). **Figure S7.** The bacterial presence within the biofilm was observed through confocal microscopy with live/dead staining in the following groups. **A** PBS-treated group. **B** ETP-treated group. **C** ASP-treated group. **D** Au NPs-treated group. **E** ASP_Au NPs-treated group. In these images, green fluorescence represents live bacteria within the biofilm, while red fluorescence indicates dead bacteria within the biofilm. **Figure S8.** Toxicity experiment in *Galleria mellonella *larvae. Survival of *Galleria mellonella *larvae (10 per group) after injection with different concentrations of ASP_Au NPs. **Figure S9.** Weight variation curve of mice infected with clinical CRE and treated with different formulations over time. **Figure S10.** Histopathological sections of major organ tissues (heart, liver, spleen, lung, kidney) in mice injected with PBS (represented in the figure as 0 μg/mL) or ASP_Au NPs. The sections were stained with H&E and observed under a microscope. The injection dosage and time correspond to the acute intraperitoneal infection model in mice. **Figure S11.** Hematological analysis of mice injected with PBS (represented in the figure as 0 μg/mL) or ASP_Au NPs, reflecting changes in major inflammatory cells in the blood. WBC represents white blood cell count, and NEUT represents neutrophil count. **Figure S12.** Biochemical analysis of mice injected with PBS (represented in the figure as 0 μg/mL) or ASP_Au NPs. **A** Reflects liver function with 8 parameters. **B** Reflects cardiac enzyme profile with 4 parameters. **C** Reflects renal function with 3 parameters. The abbreviations in the figure are as follows. ALT: alanine aminotransferase; *AST* aspartate aminotransferase, *T-BIL* total bilirubin, *D-BIL* direct bilirubin, *ALB* albumin, *ALP* alkaline phosphatase, *γ-GT* γ-glutamyl transpeptidase, *TBA* total bile acid, *BUN* blood urea nitrogen, *CREA* serum creatinine, *UA* uric acid, *CK* creatine kinase, *CK-MB* creatine kinase-MB, *LDH* lactate dehydrogenase, *LDH-1* lactate dehydrogenase-1. **Table S1.** Binding efficiencies of NAS on AuNPs. **Table S2.** Mechanism of carbapenem resistance and antimicrobial susceptibility of the IPM, MEM and ETP against 32 strains used in this study. **Table S3.** Antimicrobial susceptibility of ASP_Au NPs before and after using quercetin against the 6 clinical isolates used in this study. **Table S4.** Antimicrobial susceptibility of ASP and Au NPs single or in combination against the 6 clinical isolates used in this study. **Table S5.** Reference range of biochemical indicators in mice. **Table S6.** Main materials used in this study and the corresponding manufacturers. **Table S7.** Primers used to amplify mRNAs via RT-qPCR.

## Data Availability

The data utilized and analyzed in this study are included within this article and can be obtained from the first author upon reasonable request.

## Abbreviations

CRE: Carbapenem-resistant *Enterobacteriaceae*
Au NPs: Gold nanoparticles
NAS: Non-caloric artificial sweeteners
SAC: Saccharin
SUC: Sucralose
ACE: Acesulfame
ASP: Aspartame
SAC_Au NPs: SAC-decorated Au NPs
SUC_Au NPs: SUC-decorated Au NPs
ACE_Au NPs: ACE-decorated Au NPs
ASP_Au NPs: ASP-decorated Au NPs
NAS_Au NPs: NAS-decorated Au NPs
ROS: Reactive oxygen species
DMSO: Dimethyl sulfoxide
MIC: Minimum inhibitory concentration
HAuCl_4_: Tetrachloroauric acid
NaBH_4_: Sodium borohydride
DLS: Dynamic light scattering
PDIs: Polydispersity indices
TEM: Transmission electron microscopy
PCR: Polymerase chain reaction
ETP: Ertapenem
XPS: X-ray photoelectron spectroscopy
FTIR: Fourier-transform infrared spectroscopy
SEM: Scanning electron microscopy
PBS: Phosphate-buffered saline
H&E: Hematoxylin and eosin
WBC: White blood cells
NEUT: Neutrophils
ALP: Alkaline phosphatase
CREA: Creatinine
RT-qPCR: Real time quantitative PCR
ELISA: Enzyme-linked immunosorbent assay
LPS: Lipopolysaccharide
LB: Luria Bertani
IPM: Imipenem
MEM: Meropenem
DCFH-DA: 2′,7′-Dichlorodihydrofluorescein diacetate
DiOC_2_(3): 3,3'-Diethyloxacarbocyanine iodide
RBCs: Red blood cells
DMEM: Dulbecco's modified eagle medium
FBS: Fetal bovine serum
SPF: Specific pathogen-free
SD: Standard deviation;
ANOVA: One-way analysis of variance
OPA: Ortho-pthaldehyde
PI: Propidium iodide
