# Supplementary material for: A potential strategy against clinical carbapenem-resistant Enterobacteriaceae: antimicrobial activity study of sweetener-decorated gold nanoparticles in vitro and in vivo
Source: J Nanobiotechnology. 2023 Nov 6;21:409. doi: 10.1186/s12951-023-02149-x (PMC10626710; doi:10.1186/s12951-023-02149-x)
Supplement: Supplementary file 1 — Additional file 1: Figure S1. UV-visible spectra of NaBH4-reduced Au NPs. Inside the dashed box, a comparison of the peaks between NaBH4-reduced Au NPs and NAS_Au NPs are presented. Figure S2. The size, PDI and zeta potential of NaBH4-reduced Au NPs. Figure S3. XPS analysis of Au 4f, N1s, and O1s for ASP, Au NPs, and ASP_Au NPs. A Au 4f spectrum analysis of Au NPs. B Au 4f spectrum analysis of ASP_Au NPs. C N1s spectrum analysis of ASP. D N1s spectrum analysis of ASP_Au NPs. E O1s spectrum analysis of ASP. F O1s spectrum analysis of ASP_Au NPs. Hollow dots represent raw data, blue curves represent the overall fitting curve of the data, and black curves represent the baseline. Colored curves and their corresponding peak labels are shown in the figure. Figure S4. FTIR peaks of Au NPs, ASP, and ASP_Au NPs. Figure S5. The FTIR spectra for the remaining three nano-cargos. Figure S6. PI membrane permeability assay. There was a sharp increase in fluorescence intensity at MIC concentrations (8 μg/mL). Figure S7. The bacterial presence within the biofilm was observed through confocal microscopy with live/dead staining in the following groups. A PBS-treated group. B ETP-treated group. C ASP-treated group. D Au NPs-treated group. E ASP_Au NPs-treated group. In these images, green fluorescence represents live bacteria within the biofilm, while red fluorescence indicates dead bacteria within the biofilm. Figure S8. Toxicity experiment in Galleria mellonella larvae. Survival of Galleria mellonella larvae (10 per group) after injection with different concentrations of ASP_Au NPs. Figure S9. Weight variation curve of mice infected with clinical CRE and treated with different formulations over time. Figure S10. Histopathological sections of major organ tissues (heart, liver, spleen, lung, kidney) in mice injected with PBS (represented in the figure as 0 μg/mL) or ASP_Au NPs. The sections were stained with H&E and observed under a microscope. The injection dosage and time correspond [file 12951_2023_2149_MOESM1_ESM.pdf]

Additional file

A Potential Strategy against Clinical Carbapenem-Resistant *Enterobacteriaceae*: Antimicrobial Activity Study of Sweetener-Decorated Gold Nanoparticles *in Vitro* and *in Vivo*

Haifeng Liu[a], Zeyu Huang[a], Huanchang Chen[a], Ying Zhang[a], Pingting Yu[a], Panjie Hu[b], Xiaotuan Zhang[a], Jianming Cao[a*], Tieli Zhou[a*]

[a] Department of Clinical Laboratory, The First Affiliated Hospital of Wenzhou Medical University; Key Laboratory of Clinical Laboratory Diagnosis and Translational Research of Zhejiang Province, Wenzhou, Zhejiang, China

[b] School of Laboratory Medicine and Life Science, Wenzhou Medical University, Wenzhou, Zhejiang, China

* Corresponding author

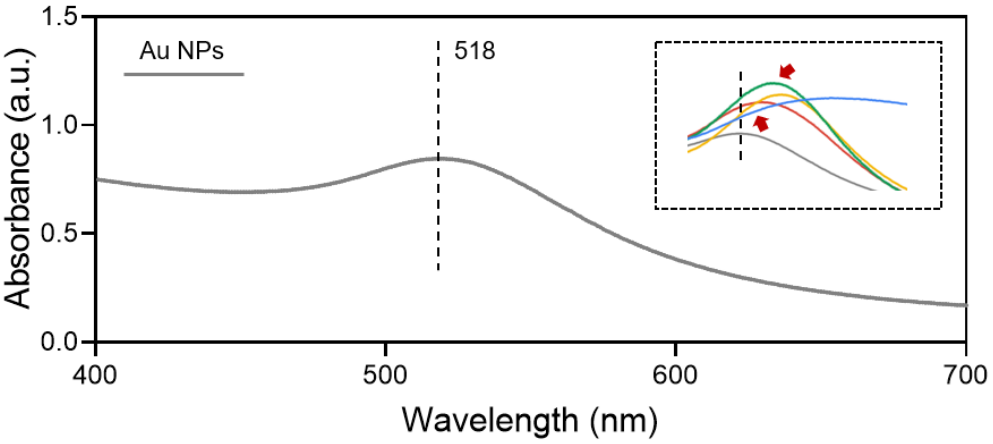

Figure S1. UV-visible spectra of $NaBH_4$-reduced Au NPs. Inside the dashed box, a comparison of the peaks between $NaBH_4$-reduced Au NPs and NAS_Au NPs are presented.

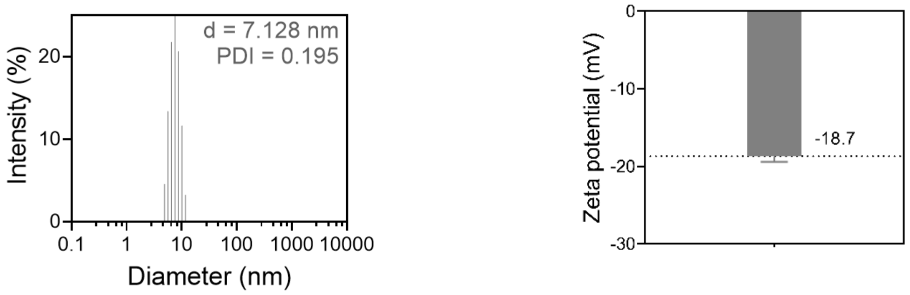

Figure S2. The size, PDI and zeta potential of $NaBH_4$-reduced Au NPs.

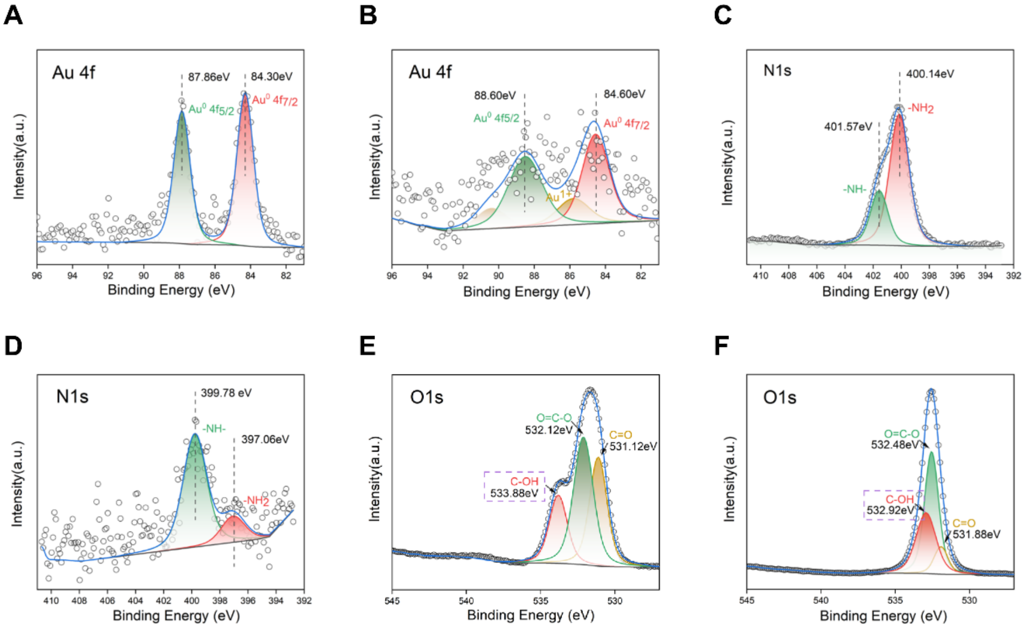

Figure S3. XPS analysis of Au 4f, N1s, and O1s for ASP, Au NPs, and ASP_Au NPs. (A) Au 4f spectrum analysis of Au NPs. (B) Au 4f spectrum analysis of ASP_Au NPs. (C) N1s spectrum analysis of ASP. (D) N1s spectrum analysis of ASP_Au NPs. (E) O1s spectrum analysis of ASP. (F) O1s spectrum analysis of ASP_Au NPs. Hollow dots represent raw data, blue curves represent the overall fitting curve of the data, and black curves represent the baseline. Colored curves and their corresponding peak labels are shown in the figure.

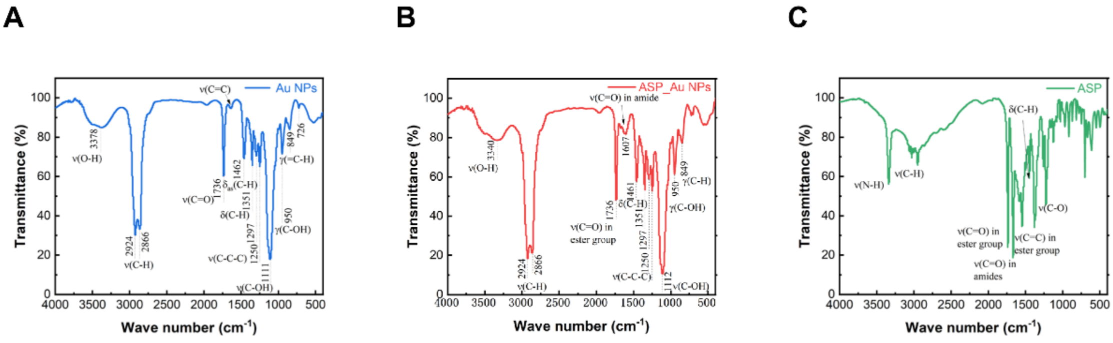

Figure S4. FTIR peaks of Au NPs, ASP, and ASP_Au NPs.

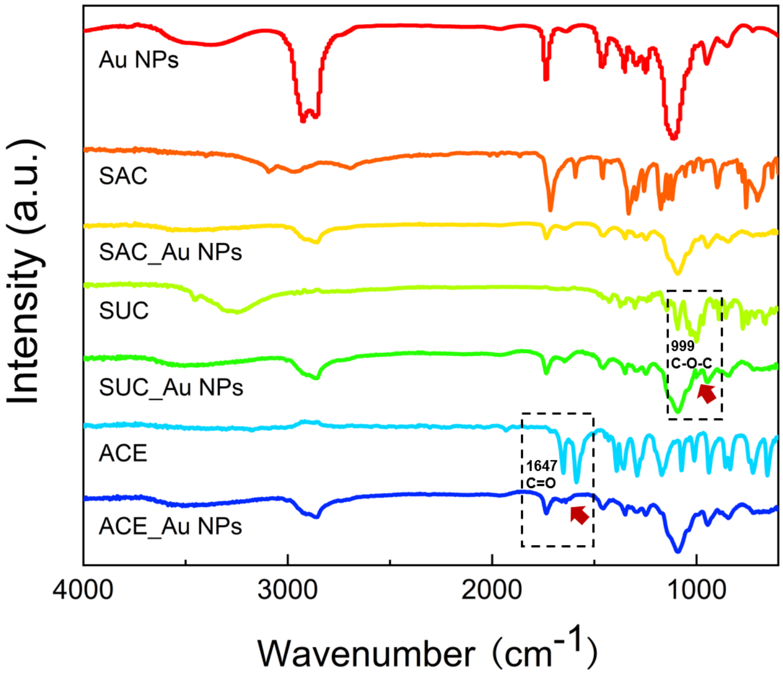

Figure S5. The FTIR spectra for the remaining three nano-cargos.

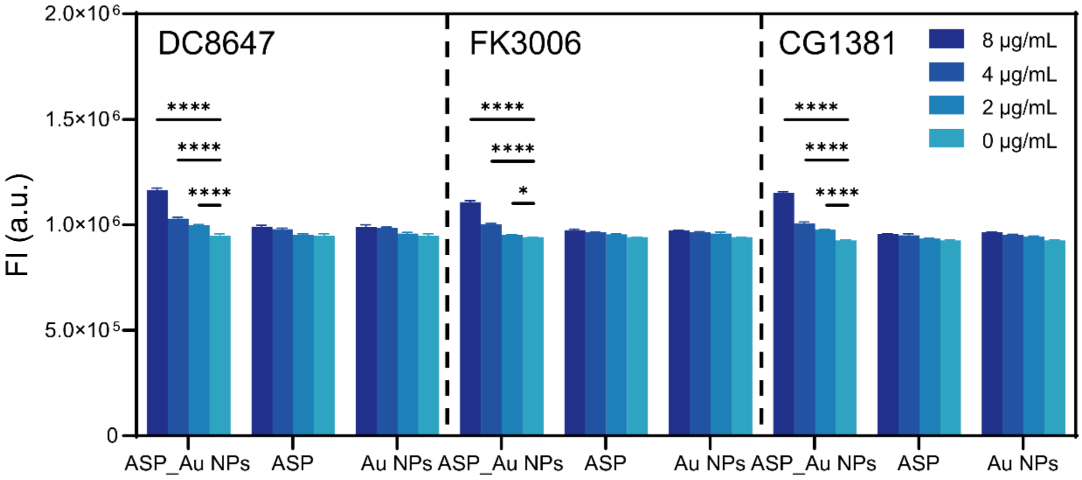

Figure S6. PI membrane permeability assay. There was a sharp increase in fluorescence intensity at MIC concentrations (8 μg/mL) .

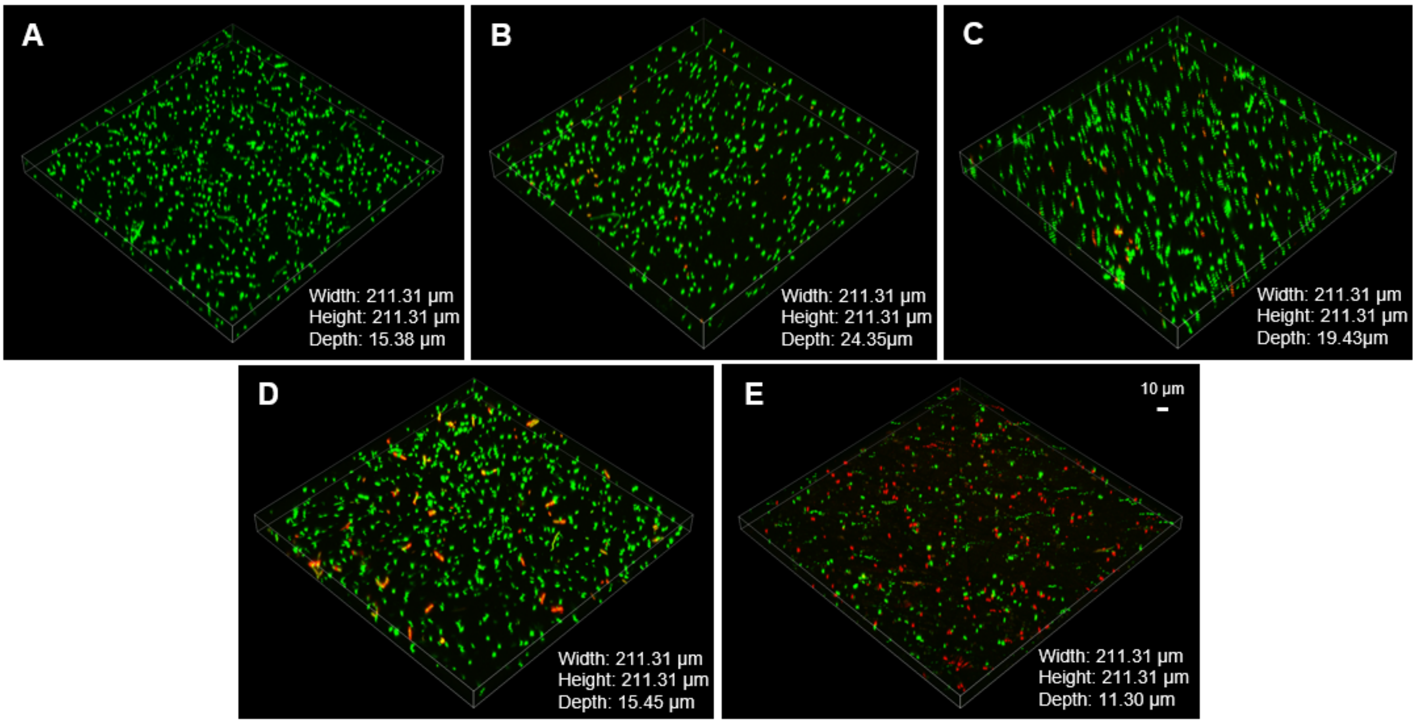

Figure S7. The bacterial presence within the biofilm was observed through confocal microscopy with live/dead staining in the following groups. (A) PBS-treated group. (B) ETP-treated group. (C) ASP-treated group. (D) Au NPs-treated group. (E) ASP_Au NPs-treated group. In these images, green fluorescence represents live bacteria within the biofilm, while red fluorescence indicates dead bacteria within the biofilm.

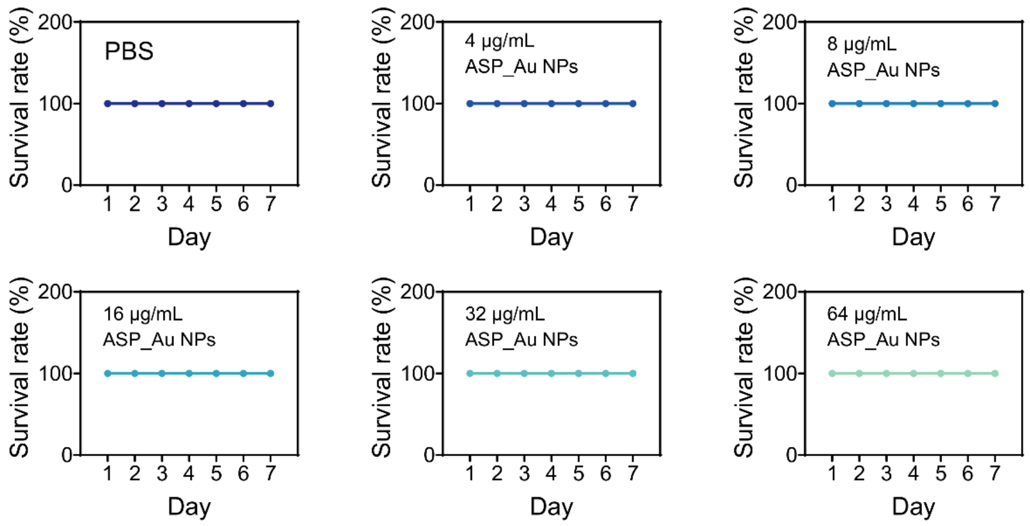

Figure S8. Toxicity experiment in *Galleria mellonella* larvae. Survival of *Galleria mellonella* larvae (10 per group) after injection with different concentrations of ASP_Au NPs.

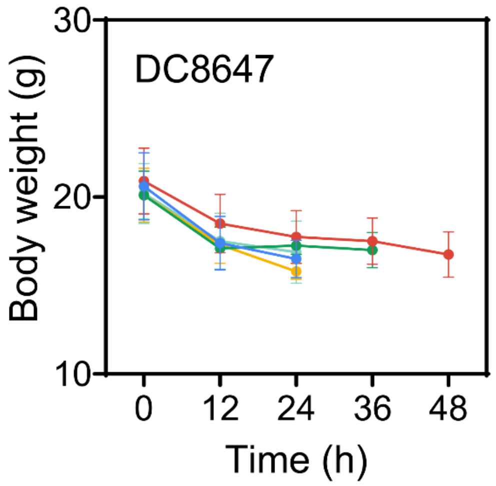

Figure S9. Weight variation curve of mice infected with clinical CRE and treated with different formulations over time.

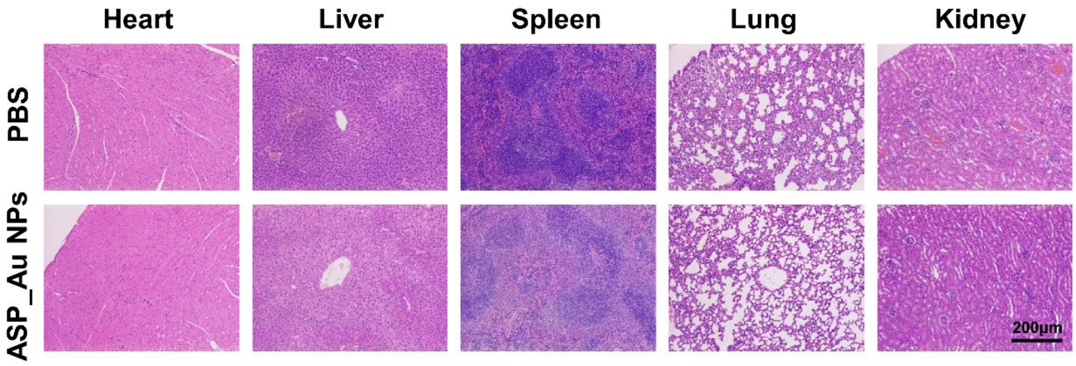

Figure S10. Histopathological sections of major organ tissues (heart, liver, spleen, lung, kidney) in mice injected with PBS (represented in the figure as 0 μg/mL) or ASP_Au NPs. The sections were stained with H&E and observed under a microscope. The injection dosage and time correspond to the acute intraperitoneal infection model in mice.

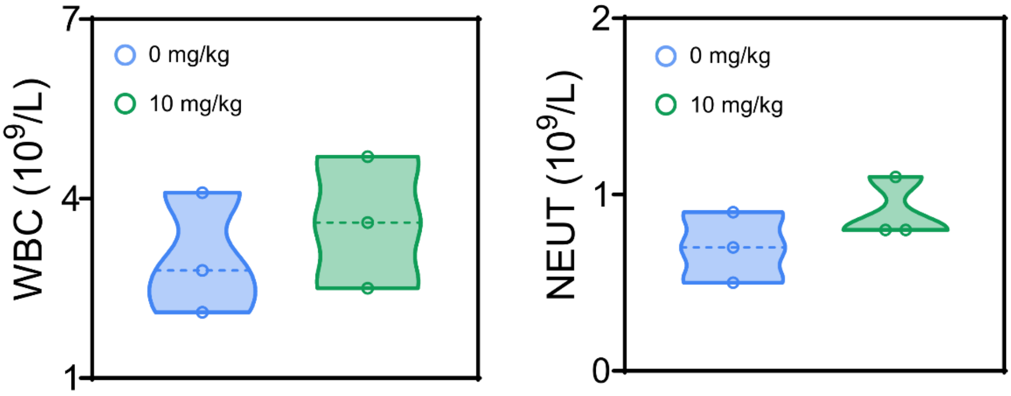

Figure S11. Hematological analysis of mice injected with PBS (represented in the figure as 0 μg/mL) or ASP_Au NPs, reflecting changes in major inflammatory cells in the blood. WBC represents white blood cell count, and NEUT represents neutrophil count.

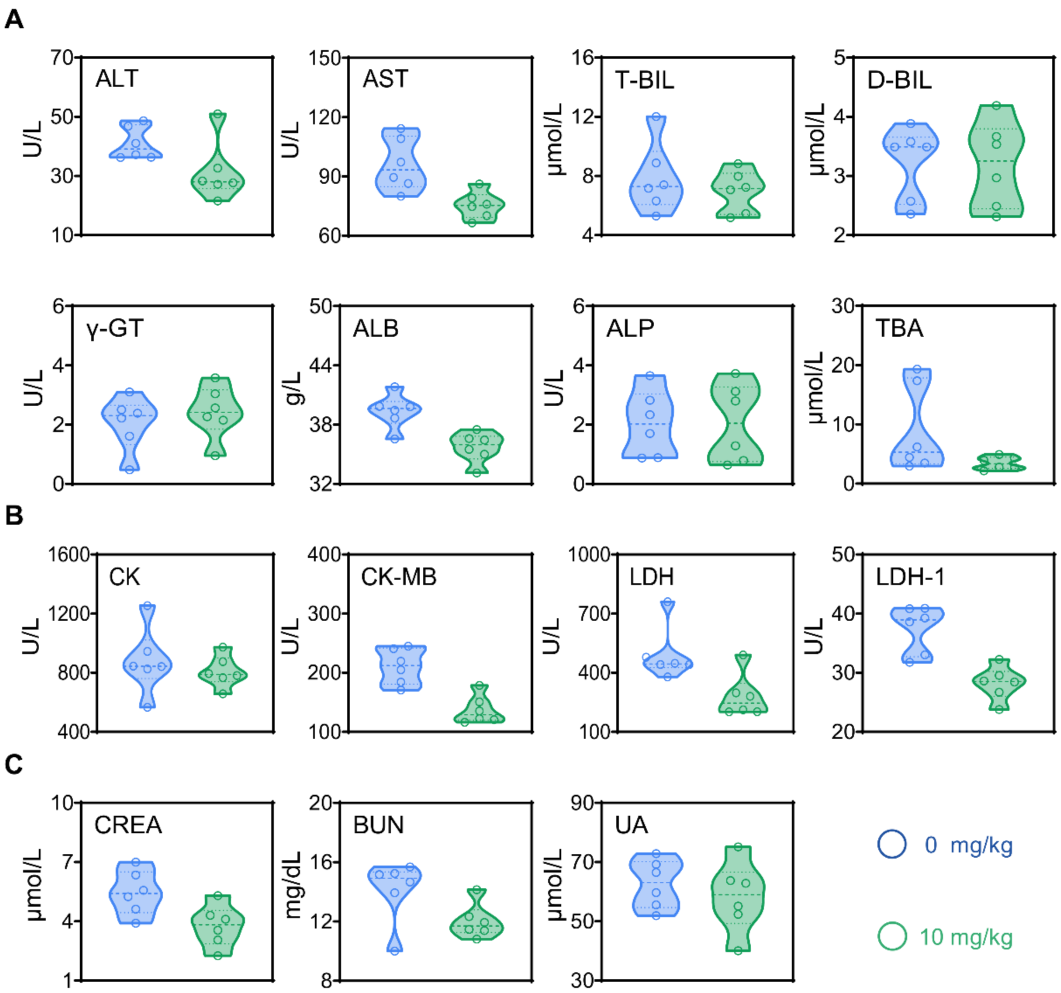

Figure S12. Biochemical analysis of mice injected with PBS (represented in the figure as 0 μg/mL) or ASP_Au NPs. (A) Reflects liver function with 8 parameters. (B) Reflects cardiac enzyme profile with 4 parameters. (C) Reflects renal function with 3 parameters. The abbreviations in the figure are as follows. ALT: alanine aminotransferase; AST: aspartate aminotransferase; T-BIL: total bilirubin; D-BIL: direct bilirubin; ALB: albumin; ALP: alkaline phosphatase; γ-GT: γ-glutamyl transpeptidase; TBA: total bile acid; BUN: blood urea nitrogen; CREA: serum creatinine; UA: uric acid; CK: creatine kinase; CK-MB: creatine kinase-MB; LDH: lactate dehydrogenase; LDH-1: lactate dehydrogenase-1.

Table S1. Binding efficiencies of NAS on AuNPs.

Different Au NPs	Relative molar ratio (NAS : Au)	Fluorescence intensity (a.u)		Conjugation efficiency (%)	Average conjugation efficiency (%)
		Conjugate Front	Conjugate After		
SAC_Au NPs	1:1	21977690	9150024	41.63	41.67
		21850286	9140222	41.83	
		21877696	9090799	41.55	
SUC_Au NPs	1:1	15189762	5002106	32.93	32.99
		15358828	5070901	33.02	
		15397770	5084144	33.02	
ACE_Au NPs	1:1	19852298	9080899	45.74	46.93
		19348502	9023710	46.64	
		19143154	9269727	48.42	
ASP_Au NPs	1:1	48024300	25739526	53.60	53.07
		48156964	25180942	52.29	
		48686552	25965126	53.33	

Table S2. Mechanism of carbapenem resistance and antimicrobial susceptibility of the IPM, MEM and ETP against 32 strains used in this study.

Species	Strains	Antimicrobial resistance mechanism	MIC (μg/mL)		
			IPM	MEM	ETP
E. coli	DC2003	CTX-M-1, CTX-M-9, OmpF	32	64	256
	DC5113	KPC-2, TEM, CTX-M-1, CTX-M-9	8	16	128
	DC5128	CTX-M-1, CTX-M-9,OmpC, OmpF, NDM	32	64	256
	DC5293	TEM, SHV	0.25	64	128
	DC6856	KPC-2, CTX-M-1, CTX-M-9, OmpF	8	16	128
	DC7114	NDM, TEM, CTX-M-1, OmpC	16	32	128
	DC7706	NDM, TEM, CTX-M-1, CTX-M-9, OmpF	32	64	128
	DC8647	NDM-1	16	32	128
	DC10694	CTX-M-1, CTX-M-9, TEM, SHV	0.5	0.5	256
	DC11722	KPC-2, OXA-1, CMY-42	32	128	512
	ATCC25922	β-lactamase negative	<0.25	<0.25	<0.25
K. pneumoniae	FK2836	KPC-2, IMP	128	64	256
	FK3006	KPC-2, IMP	32	128	256
	FK3020	IMP	32	128	256
	FK6709	KPC-2, OXA-23	32	64	256
	FK6724	KPC-2, OXA-23	32	16	128
	FK7079	CTX-M-9, SHV, TEM	8	256	128
	FK7112	KPC-2, OmpK37mutation	64	128	512
	FK7513	NDM-5	32	64	128
	FK8696	KPC-33, CTX-M-9, SHV,TEM	0.25	2	128
	FK9283	NDM-1	8	128	64
	ATCC700603	SHV-18, OXA-2, OmpK35 and OmpK37 mutations, TEM-1	0.25	0.25	0.5
E. cloacae	CG648	KPC-2, AmpC	16	32	32

CG1038	KPC-2, AmpC	8	32	128
CG1181	SHV, TEM, CTX-M-1, CTX-M-9, CTX-M-14, impermeability	2	<0.25	128
CG1212	TEM, CTX-M-14, impermeability, efflux pump	1	1	64
CG1249	AmpC, impermeability	0.5	1	128
CG1257	IMP	32	4	64
CG1330	NDM-1	8	16	128
CG1381	OXA-23	32	32	64
CG1737	NDM-5	32	64	128
CG1813	IMP, efflux pump	8	16	128

Table S3. Antimicrobial susceptibility of ASP_Au NPs before and after using quercetin against the 6 clinical isolates used in this study.

Strains	ASP_Au NPs (MIC, μg/mL)	
	Before	After
DC8647	8	≥256
DC11722	16	≥256
FK3006	8	≥256
FK7513	8	≥256
CG1330	4	≥256
CG1381	8	≥256

Table S4. Antimicrobial susceptibility of ASP and Au NPs single or in combination against the 6 clinical isolates used in this study.

Strains	Monotherapy (MIC, μg/mL)		Combination (MIC, μg/mL)	
	ASP	Au NPs	ASP	Au NPs
DC8647	≥256	≥256	≥256	≥256
DC11722	≥256	≥256	≥256	≥256
FK3006	≥256	≥256	≥256	≥256
FK7513	≥256	≥256	≥256	≥256
CG1330	≥256	≥256	≥256	≥256
CG1381	≥256	≥256	≥256	≥256

Table S5. Reference range of biochemical indicators in mice.

Testing items	Indicators	Reference range	Unit
Liver function	ALT	10.06-96.47	U/L
	AST	36.31-235.48	U/L
	T-BIL	6.09-53.06	μmol/L
	D-BIL	0.45-33.89	μmol/L
	ALB	21.22-39.15	g/L
	ALP	22.52-474.35	U/L
	γ-GT	0-7.78	U/L
	TBA	0-8.51	μmol/L
Renal function	BUN	10.81-34.74	mg/dL
	CREA	10.91-85.09	μmol/L
	UA	44.42-224.77	μmol/L
Myocardial enzymes	CK	0-2070.55	U/L
	CK-MB	0-1500	U/L
	LDH	157.41-899.72	U/L
	LDH-1	0-37.07	U/L

Table S6. Main materials used in this study and the corresponding manufacturers.

Materials	Manufacturer
$HAuCl_4 \cdot 3H_2O$ (48~50% Au basis)	Macklin (Shanghai, China)
Tween 80	Solarbio (Beijing, China)
Triethylamine	Wenzhou Jinshan Chemical Reagent Instrument Co., Ltd. (Zhejiang, China)
Dialysis filter membranes	Solarbio (Beijing, China)
SAC	Macklin (Shanghai, China)
SUC	Macklin (Shanghai, China)
ACE	Macklin (Shanghai, China)
ASP	Macklin (Shanghai, China)
Quercetin	MedChemExpress (China)
Glycerol	Thermo Fisher Scientific (America)
$DiOC_2(3)$	Maokang Biology (Shanghai)
NaBH4	Macklin (Shanghai, China)
CCK-8	Solarbio (Beijing, China)
Propidium iodide	Solarbio (Beijing, China)
SYTO 9	Thermo Fisher Scientific (America)
Boric acid	Aladdin (Shanghai, China)
OPA	Aladdin (Shanghai, China)
NaOH	Aladdin (Shanghai, China)
ROS assay kit	Beyotime (Shanghai, China)
Protein detection kits	Boyun (Shanghai, China)
RevertAid First Strand cDNA Synthesis Kit	Thermo Fisher Scientific (America)
Tli RNaseH Plus	Takara (Japan)
Cyclophosphamide	Shanghai Yuanye Bio-Technology Co., Ltd. (Shanghai, China)
2.5% glutaraldehyde	Servicebio (Hubei, China)

Table S7. Primers used to amplify mRNAs via RT-qPCR.

**Gene**	**Forward primer (5′ - 3′)**	**Reverse primer (5′ - 3′)**
IL-1β	GCAACTGTTCCTGAACTCAACT	ATCTTTTGGGGTCCGTCAACT
TNF-α	GAGTCCGGGCAGGTCTACTTT	CAGGTCACTGTCCCAGCATCT
β-actin	AGCCATGTACGTAGCCATCC	CTCTCAGCTGTGGTGGTGAA
